# A Brief Review of Recent Superconductivity Research at NIST

**DOI:** 10.6028/jres.094.018

**Published:** 1989

**Authors:** D. R. Lundy, L. J. Swartzendruber, L. H. Bennett

**Affiliations:** National Institute of Standards and Technology, Gaithersburg, MD 20899

**Keywords:** ceramics, copper oxides, cryogenic engineering, crystal structure, electronic structure, materials science, measurement science, overview, perovskites, superconductivity

## Abstract

A brief overview of recent superconductivity research at NIST is presented. Emphasis is placed on the new high-temperature oxide superconductors, though mention is made of important work on low-temperature superconductors, and a few historical notes are included. NIST research covers a wide range of interests. For the new high-temperature superconductors, research activities include determination of physical properties such as elastic constants and electronic structure, development of new techniques such as magnetic-field modulated microwave-absorption and determination of phase diagrams and crystal structure. For the low-temperature superconductors, research spans studying the effect of stress on current density to the fabrication of a new Josephson junction voltage standard.

## 1. Introduction

Research in superconductivity at NIST has a long history, in part because of its important applications to measurements and materials science. The recent discovery of materials with unexpectedly high superconducting transition temperatures has brought renewed interest in the science and technology of superconductors throughout the world. The purpose of this paper is to briefly review recent activities at NIST in this field and to discuss current usage and future possibilities for both conventional and high temperature superconductors. Some references will be made to historical superconductivity contributions at NIST but a complete review is not attempted.

## 2. Background

The field of superconductivity began with the discovery by H. Kamerlingh-Onnes in 1911 that mercury wire at 4.2 K had zero electrical resistance. Zero resistance implied transmission of current at any distance with no losses, the production of large magnetic fields, or—because a superconducting loop could carry current indefinitely—storage of energy. These applications were not realized because, as was quickly discovered, the superconductors reverted to normal conductors at a relatively low current density, called the critical current density, *J*_c_, or in a relatively low magnetic field, called the critical field, *H*_c_. In 1916, Silsbee, at the National Bureau of Standards, hypothesized [1] that the critical current for a superconducting wire was equal to that current which gave the critical field at the surface of the wire. The reason for this behavior was not made clear until the discovery [2] of the Meissner effect in 1933.

The discovery and development, in the 1950s and 1960s, of superconductors which can remain superconducting at much higher fields and currents made practical the production of useful superconducting magnets (see table 1). Such high-field superconductors, which exhibit two critical fields designated *H*_C1_ and *H*_C2_, are called type-II. In 1950, another NBS scientist, E. Maxwell, was the discoverer [3] of the isotope effect, which was also independently discovered by Serin et al. [4]. This experimental observation was an important key to theoretical explanations of the mechanism of superconductivity. In the isotope effect, the critical temperature for many superconductors depends on the isotopic mass, indicating that lattice vibrations are involved in the superconductivity, and that the attractive coupling between electrons is through the lattice vibrations (i.e., phonon-mediated). The discovery [5] of the Josephson effect in 1962 opened up exciting potential for the use of superconductors in measurement science and in high-speed electronic devices.

Until 1986, the highest critical temperature obtained for any superconductor was only 23.2 K. This meant that superconductors had to be cooled by liquid helium—an expensive and sometimes unreliable process. Consequently, many potential applications were not commercially viable. In addition, most scientists had come to regard superconductivity as a mature field with little possibility for any significant increase in critical temperatures. All this suddenly changed with the discovery by K. A. Bednorz and J. G. Müller of high-temperature superconductivity.

**Figure 1:**
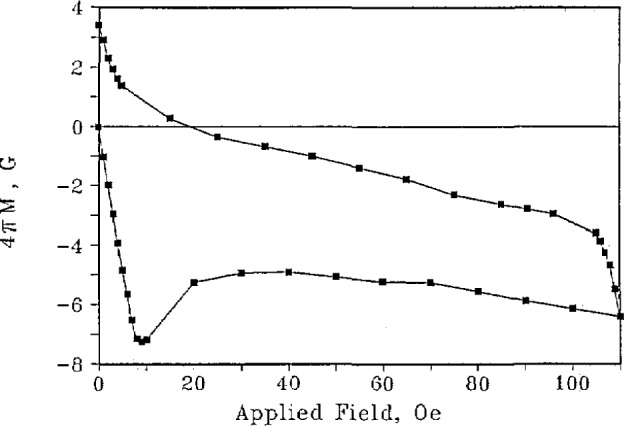
Partial hysteresis loop of SrTiO_3_ obtained at 0.15 K using a ballistic galvanometer. The shape of the loop indicates that SrTiO_3_ is a type-II superconductor. (Unpublished data courtesy of J. F. Schooley.)

In April 1986, Müller and Bednorz published a paper [6] on the possible existence of superconductivity in a ceramic material, La-Ba-Cu-O, with a superconducting transition temperature, *T*_c_, of 30 K, the first increase since 1973. Their discovery was the result of several years of extensive investigations on metal oxides, some of which had earlier been shown to be superconducting. It is noteworthy that superconductivity in oxides had been known for many years. In fact the first oxide superconductor was discovered at NIST. In 1964, Cohen predicted [7] that, based on the Bardeen-Cooper-Schrieffer (BCS) theory [8], semiconductors could become superconductors. At this time a metal-oxide semiconductor—SrTiO_3_—being investigated at NIST seemed to have the characteristics postulated by Cohen as necessary for superconductivity. Kahn and Leyendecker [9] had determined the energy band structure of SrTiO_3_ and Frederikse et al. [10] had determined the density of states in the conduction band. Schooley et al. [11] found superconductivity below 0.3 K in reduced SrTiO_3_ in 1964; this oxide was demonstrated to be a type-II superconductor by Ambler et al. [12] in 1966 (see fig. 1). *T*_c_ was only 0.3 K. however. Though substituting Ca and Ba for Sr [13] raised the *T*_c_ to 0.5 K, the low critical temperatures limited general interest in these materials.

According to Müller and Bednorz [6], their research was influenced by the French work on the La-Ba-Cu-0 system [14]. However, the French scientists were not looking for superconductivity. When researchers from the University of Tokyo [15] confirmed the findings of Müller and Bednorz, the era of “High-Temperature Superconductivity” was ushered in.

The end of 1986 and the beginning of 1987 was marked by synthesis of rare-earth metal oxides of increasingly higher *T*_c_, culminating with the discovery [16] of the Y-Ba-Cu-O (YBCO) superconductor with a *T*_c_ of 93 K. This was a significant breakthrough because the material was superconducting in liquid nitrogen (boiling point=77 K). Nitrogen is much more abundant than helium, much less expensive, and liquid nitrogen cryogenic systems are less complex than systems using helium refrigeration. One application which could benefit from nitrogen cooling is the development of hybrid microelectronic technology (semiconductor-superconductor devices)—both gallium arsenide and silicon can be tailored to perform better at liquid nitrogen temperatures.

The ease of making YBCO permitted its investigation by many laboratories. In fact, a number of high school students synthesized it for use in science fair projects. At various times researchers reported *T*_c_’s greater than 100 K—some reported superconductivity at room temperature and above. These observations were not confirmed; many of the results were irreproducible or the samples were not stable. At the end of 1987, the highest *T*_c_ stood at 95 K. In February 1988, Japanese, Chinese, and U.S. researchers found superconductivity in copper-containing oxides without rare earths. These new non-rare-earth containing superconductor materials incorporate either bismuth or thallium. Compounds containing the latter have a confirmed *T*_c_ of ≈ 127 K. These new high-temperature superconductors containing bismuth or thallium may have some advantages over the superconductors containing rare-earths. Since the critical current density increases as *T/T*_c_ decreases, a *T*_c_ far above the operating temperature of liquid nitrogen (77 K) is advantageous. Furthermore, the new materials are more stable than the rare earth superconductors; they do not lose oxygen or react with water.

In addition to trying to develop new high-*T*_c_ materials, researchers also were trying to fabricate materials with improved critical current densities (*J*_c_). Current densities as high as 10^5^–10^6^ A/cm^2^ may be needed for applications such as magnets, motors, and electronic components. The high-temperature superconductors are ceramics and have all the brittleness problems associated with non-superconducting ceramics. In addition, *J*_c_ is not an intrinsic property of superconductors but is a function of the processing procedure. The rare-earth superconductors also have highly directional properties. Therefore, a crucial problem is to fabricate the material into a useful shape and still have sufficiently high *J*_c_ and mechanical strength for practical applications.

Single crystal films of YBCO have current densities above a million A/cm^2^. However, results for bulk polycrystalline materials are orders of magnitude less. Recently, researchers have grown non-oriented polycrystalline thallium films with *J*_c_ in the millions [17]. Novel processing techniques such as explosive compaction, rapid solidification and laser ablation are currently being explored.

NIST personnel have been actively engaged in fabricating and characterizing high-*T*_c_ superconducting materials. This paper is a brief synopsis of their wide-ranging activities. Some mention of significant work in other superconductors will also be made.

## 3. Crystal Structure

**Figure 2:**
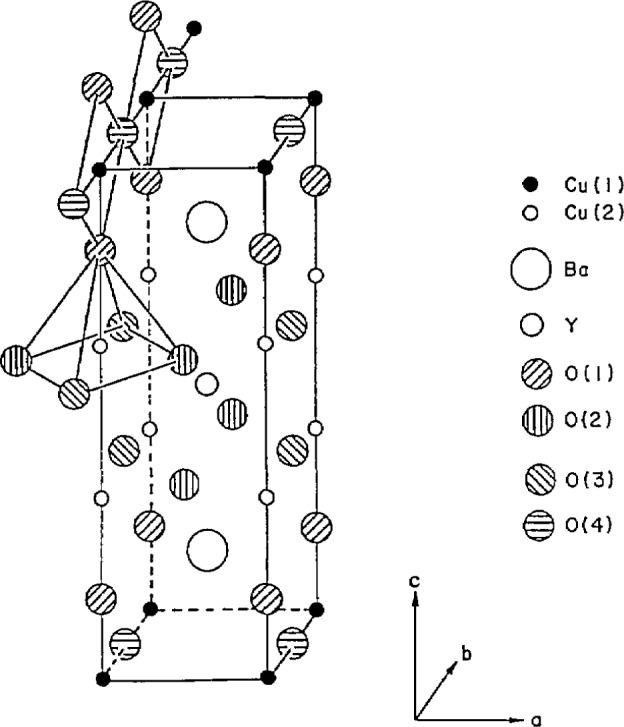
Crystal structure of YBCO as determined from neutron diffraction showing location of the four O sites, two Cu sites and single Ba and Y sites [21].

As was true for the previously mentioned SrTiO_3_, the superconducting YBCO phase is a distorted perovskite [18]. Ideal perovskites have the form ABX_3_, where A and B are metallic cations and the X atoms are non-metallic anions. In superconducting yttrium barium copper oxide, the structure (fig. 2) is a defect perovskite of the form YBa_2_Cu_3_O_7−_*_x_* (YBCO). Oxygen and oxygen vacancies are the key to the superconductivity. One widely-used method for refining the structure of YBCO is neutron diffraction because x-rays are not sensitive to oxygen atoms.

YBa_2_Cu_3_O_7−_*_x_* with *x*=0, 0.2, 0.5, and 1 was studied [19–22] using the neutron diffraction facilities of the NIST reactor. YBa_2_Cu_3_O_7_ and YBa_2_Cu_3_O_6.8_ are orthorhombic and superconducting, and are characterized by Cu-O chains along the *b* axis. In the O_7_ material, oxygen atoms occupy 4 different sites with 0(4) forming chains along the *b*-axis direction of the orthorhombic cell. The environments of the barium atoms and the copper atoms located at (000), (00z) change significantly with the amount of oxygen in the cell. YBa_2_Cu_3_O_6_, which is tetragonal and a semiconductor, is derived from YBa_2_Cu_3_O_7_ by removing oxygen along the *b* axis. While all the oxygen sites are occupied in the O_7_ material, in the O_6.8_, there are oxygen vacancies located in the chains. The superconducting *T*_c_ is reduced for deviations from O_7_, indicating that oxygen vacancies disrupt conduction pathways.

**Figure 3:**
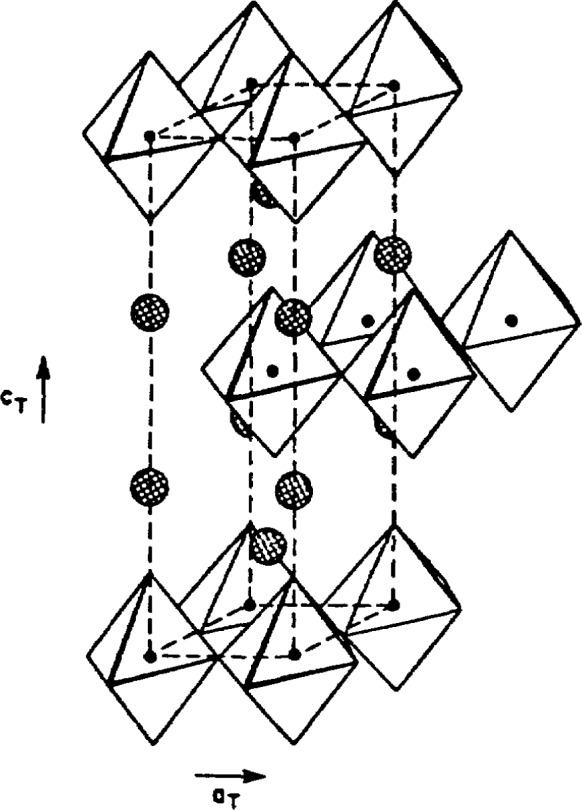
Generalized view of the highly two-dimensional tetragonal La_1.85_Sr_0.15_CuO_4_ structure. On the scale of the figure, the orthorhombic and the tetragonal structures are not distinguishable except that the orthorhombic unit cell is twice the area in the plane perpendicular to *c.* The large shaded areas are La and Sr atoms; the small circles, Cu. Oxygen atoms are at the vertices of polyhedra [23].

The crystal structure of a strontium analog of La-Ba-Cu-O, La_1.85_Sr_0.15_Sr_0.15_CuO_4_ was also examined by neutron diffraction [23]. This highly two-dimensional structure, shown in figure 3, was found to be tetragonal at ambient temperature, but became orthorhombic at 200 K, resulting in the buckling of the Cu-O planes.

## 4. Impurity Effects

One of the outstanding questions in high-T_c_ YBa_2_Cu_3_O_7_ superconductors is the relative importance of the Cu-O_2_ planes [Cu(2)-site] and the Cu-O chains [Cu(l)-site]. Zn and Ga [24] have been used to selectively substitute the Cu(2) and Cu(l) sites while maintaining the oxygen content near 7, as determined using neutron diffraction. These results, plus other recent work on Al, Co, and Fe substitutions, have shown that, in general, 3^+^ ions (e.g., Ga, Co, etc.) substitute predominantly for Cu^++^ on the chain sites, suppress the orthorhombic crystal distortion, and enhance the overall oxygen content above 7 due to valency effects. On the other hand, di-positive Zn^++^, which substitutes only on the “plane” sites, retains the orthorhombic structure, but rapidly destroys superconductivity with only a few percent Zn substitution. These neutron results (combined with bulk data) have demonstrated that the integrity of the planes is much more important in sustaining high-*T*_c_ superconductivity than the chains, and have also shown that the orthorhombic cell distortion is not essential for high transition temperatures.

Much of the prevailing theoretical work pertaining to the origin of the superconducting pairing in the high-*T*_c_ superconductors has focussed on a magnetic coupling of spins. YBa_2_Cu_3_O_7−_*_x_* with total oxygen content below ≈ 6.5 (i.e., *x* >0.5) has been shown to exhibit strong antiferromagnetic correlations. To examine these matters in more detail, neutron diffraction studies [25] were carried out on materials in which Co has been substituted for the Cu on the “chain” site in order to enhance the magnetic interactions. The results showed magnetic ordering temperatures near 400 K, representing more than enough energy to account for the ≈ 95 K superconducting transition temperatures. For Co concentrations of 20%, distinct ordering temperatures were found for plane (≈ 400 K) and for chain (≈ 200 K) site antiferromagnetic orderings, while for 80% substitutions, both sites ordered at the same temperature (≈ 435 K). The form of the temperature dependence of the observed magnetization revealed strong couplings, both within the planes and also between chain and plane sites.

By using the Mössbauer effect in Fe-doped rare earth-barium-copper-oxygen samples (with the rare earth being Y, Pr, or Er) the antiferromagnetic coupling correlations could be directly observed [26, 27]. Since the Fe atoms substitute for the Cu on both chain and plane sites, the antiferromagnetism present on the plane sites and the paramagnetism on the chain sites could be simultaneously observed in the Mössbauer patterns. It was also demonstrated that asymmetries in the Mossbauer spectra are the result of a preferential alignment of the crystallites that arise during the normal sample preparation process.

## 5. Synthesis

**Figure 4:**
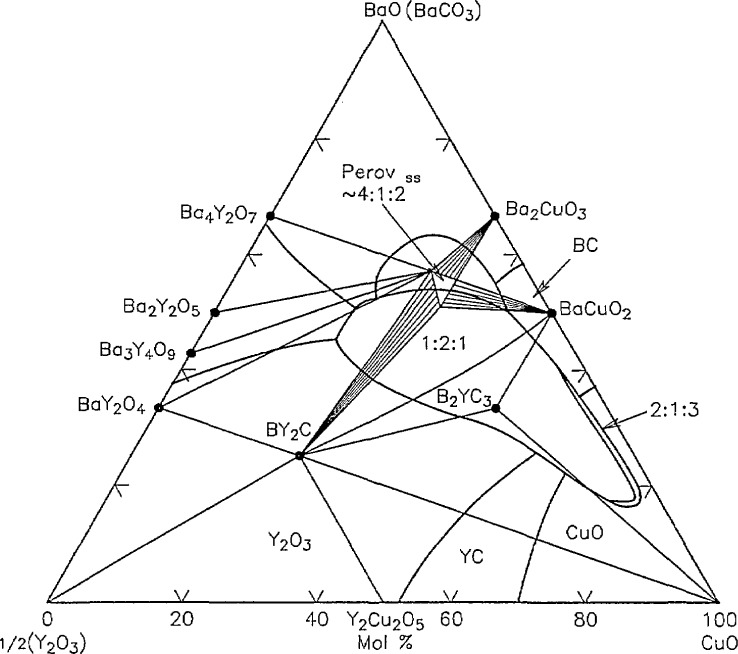
Ternary phase diagram of Y-Ba-Cu-O constructed from figures 1 and 6 of reference [37]. (Unpublished figure courtesty of R. Roth.)

Phase equilibria diagrams provide phase compositions and relationships under specific conditions. Such information is needed to characterize materials and develop synthesis procedures. Roth and his colleagues have been active in determining phase relationships for the Y-Ba-Cu-O system. Preliminary phase diagrams were constructed [28] for the binary systems BaO-1/2Y_2_O_3_; BaO-CuO; and 1/2Y_2_O_3_-Cu0*_x_*, the bounding oxide systems of the ternary, and Y-Ba-Cu-O. Nine compounds are found in the BaO-Y_2_O_3_-CuO*_x_* system [29, 30] in the temperature range 950–1000 °C. Three of the compounds, Ba_2_YCu_3_O_7−_*_x_* (the superconducting phase), BaY_2_CuO_5_ (the “green” phase which is found to exist with other phases), and Ba_3_YCu_2_O_z_ were characterized by x-ray diffraction (XRD) [31–33]. Substitutions of lanthanides for yttrium in BaY_2_O_5_ were also characterized by XRD [34]. Fourteen standard reference patterns for six high- *T*_c_ superconducting and related phases have been reported [35]. Roth [28] found that all compositions in the ternary system containing 50% BaO always showed a small amount of the green phase. A tenth phase, Ba_2_CuO_3_, which has a melting point below 950 °C was characterized by XRD [36]. Studies of phase equilibria in air [37] showed that the superconductor phase melts through a four-phase region (fig. 4) from about 950–1002 °C. This region is due to the presence of CO_2_, probably mainly in the liquid phase. The presence of CO_2_ in the superconducting phase was inferred. The transition from tetragonal to orthorhombic structure was concluded to be metastable and no large primary phase field consisting only of the superconducting phase and liquid was identified.

The substitution of SrO for BaO in the BaO:Y_2_O_3_:CuO system was studied to determine the extent of solid solution of Sr in YBCO and to identify any new phases. It was found that Sr could be substituted for Ba up to about 60%. There were no ternary compounds in the Sr-Y-Cu-O equivalent to the three ternary phases in the Ba system, but a new binary phase Sr_14_Cu_24_O_41_ was found. The SrO-CaO-CuO system was also studied as part of an investigation of the SrO-CaO-Bi_2_-O_3_-CuO system. At 950 °C, there were three extensive solid solutions at (Sr, Ca):Cu ratios of 2:1, 1:1, and 24:41. A new ternary Sr*_x_*Ca_1−_*_x_*CuO_2_ (*x*0.15) was found and a new phase, probably CaCu_2_, stable only below ∼740 °C, was identified [38, 39].

**Figure 5:**
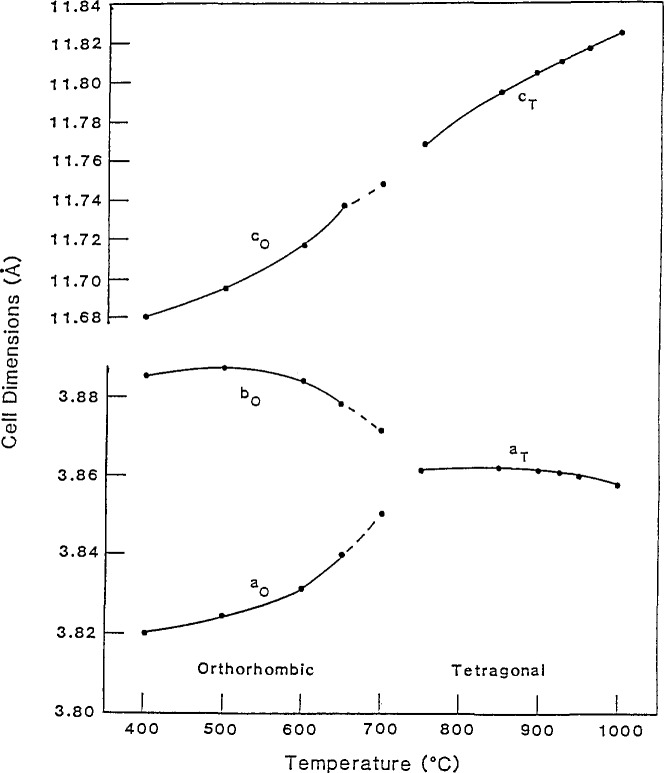
A plot of the cell dimensions of YBCO as a function of annealing temperature. As the temperature increases, the cell dimension *b*_0_ decreases while *a*_0_ increases [40].

**Figure 6:**
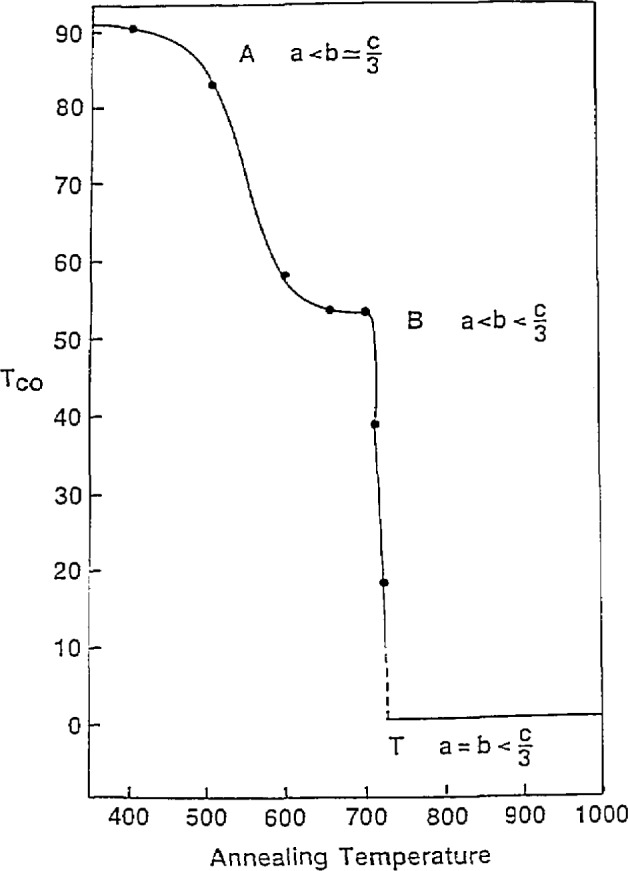
A plot of *T*_Co_ versus annealing temperature. Note the two plateaus at 91 K and 58 K [41].

Since the oxygen content in YBa_2_Cu_3_O_7__*_x_* strongly affects the superconducting and structural properties, the effects of variations in annealing (oxygenating) were studied. Single-phase samples were annealed at temperatures from 400 °C to 1000 °C [40, 41], then quenched in a liquid-nitrogen-cooled copper cold well, through which liquid-nitrogen-cooled helium gas was passed at a rapid rate. The goal was to quench in the high-temperature structures and stoichiometrics. Samples were initially examined by x-ray diffraction. It was observed that, as the annealing temperature decreased, the ceramic became more orthorhombic—going from fully tetragonal at 1000 °C to fully orthorhombic at 400 °C (fig. 5). The phase transition occurred at 708–719 °C. The dependence of cell volume on temperature was not linear, becoming substantial only at 400–650 °C, the orthorhombic region. The limiting volumes were the volume of YBa_2_Cu_3_O_7_ annealed in air and YBa_2_Cu_3_O_6_ annealed in argon. Two possible orthorhombic regions were indicated—*a* <*b* =*c*/3, and *a* <*b* <*c*/3. ac susceptibility measurements were made for samples annealed up to 708 °C (samples annealed above 750 °C showed no Meissner effect). As shown in figure 6, a plot of *T*_co_ (*T*_c_ onset) versus annealing temperature showed two plateaus— 91 and 58 K. While these might indicate two different orthorhombic phases, lack of corroborating x-ray data prevented a firm identification. YBa_2_Cu_3_O_7−_*_x_* was also examined by thermogravimetric analysis (TGA) and differential scanning scanning calorimetry (DSC). TGA results also gave indications of two regions. DSC/TGA analysis showed a thermal event when YBa_2_Cu_3_O_7___x_ was heated to 900 K which might be the result of microhomogeneities or of discontinuities in the oxygen vacancy ordering [42]. DSC studies of this material [43] showed that the phase transition does not associate with an enthalpy change, a characteristic of second-order transitions. Based on this and the x-ray data, the phase transition appears to be a second order, order-disorder type. TGA was also used to determine the oxygen diffusion coefficient of YBCO [44]. The oxygen was found to diffuse faster than in some insulating oxides, such as A1_2_O_3_, but slower than in oxides which have been classed as oxygen conductors. The diffusion constant plotted against 1/*T* is linear from 400–600 K. From 600–750 K, it is not linear and only weakly dependent on temperature, which may be due to structural phase changes.

Structural phase transitions of Ba_2_RCu_3_O_6+_*_y_* (where R=Sm, Gd, or Er) were studied [45] to determine the effect of the span of radii and the magnetic properties of Gd and Er. Samples were annealed at 400–1000 °C and quenched in a liquid-nitrogen-cooled copper well. The x-ray spectra were similar to that of YBCO. The orthorhombic-tetragonal transition always occurred between 625–770 °C. The Gd compounds showed an increase in the *c* axis due to oxygen vacancies, as in YBCO. The rare-earth elements with smaller radii stabilized the orthorhombic phase to a higher temperature. The phase transformations are apparently second order and may involve two orthorhombic regions which correspond to two *T*_c_ plateaus—one at 92 K and one between 52 and 60 K. These regions have the same general structure, but different oxygen distributions corresponding to ordered and disordered modifications within the orthorhombic structure. No obvious plateau was detected for the Gd compounds however. TGA also identified plateaus—three apparently single-phase regions for Sm, Y, and Er and two for Gd. The fact that the literature *T*_c_ values are about the same for all the compounds suggests that the superconducting electrons are not strongly associated with the rare-earth elements [46].

**Figure 7:**
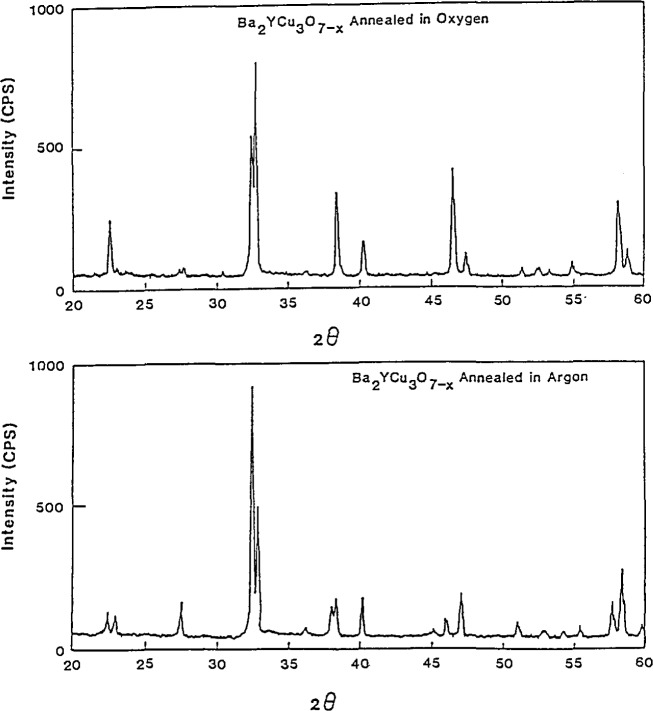
Effect of oxygen on the structure of YBCO. a) x-ray diffraction pattern of YBCO annealed in air (orthorhombic). b) x-ray diffraction pattern of YBCO annealed in argon (tetragonal). The most striking features are the intensity reversal of the two sets of doublets at around 32–33° and 57–60°, and the shifting of positions of corresponding peaks which indicates different cell dimensions [47].

The effect of annealing atmosphere (fig. 7) was also studied [47, 48]. Samples annealed and cooled in oxygen were found to have sharper superconducting transitions than those annealed and cooled in air. They also had sharper diffraction peaks. The broadening is due to crystallite size differences and microstrain/chemical inhomogeneity which can originate from twinning, anisotropic thermal expansion, and oxygen vacancies. Thermal analysis determined that the maximum oxygen content is obtained by annealing at 450 °C, or slightly above, and that the oxygen loss is reversible [43].

It is clear from these results that the processing parameters must be carefully controlled to yield the desired material. There is an additional concern. While it is known that exposure to water can destroy the superconducting ability of YBa_2_Cu_3_O_7_ it also has been found that acetone can be deleterious [49]. To obtain dense, strong ceramics, the powders are often milled to a small size before sintering. When an acetone slurry is used, a non-superconducting tetragonal phase can be formed if the slurry is dried at 200 °C. The superconducting orthorhombic phase can be restored by annealing at 950 °C in O_2_.

It may be possible to avoid the grinding steps altogether by employing chemical synthesis. Four systems—the coprecipitation of yttrium, barium, and copper hydroxy-carbonates; the hydrolysis of yttrium, barium, and copper alkoxides in ethanol/toluene; the reaction of barium and yttrium alkoxides with Cu(OH)_2_; and the hydrolysis of yttrium, barium, and copper alkoxides in methoxyethanol/ethanol were studied [50]. All the systems showed BaCO_3_, CuO, and Y_2_O_3_ when heated at 400–600 °C. The samples must then be heated to 800–950 °C to obtain YBa_2_Cu_3_O_7−_*_x_* and subsequently annealed in oxygen at 450–600 °C to obtain superconductivity.

**Figure 8:**
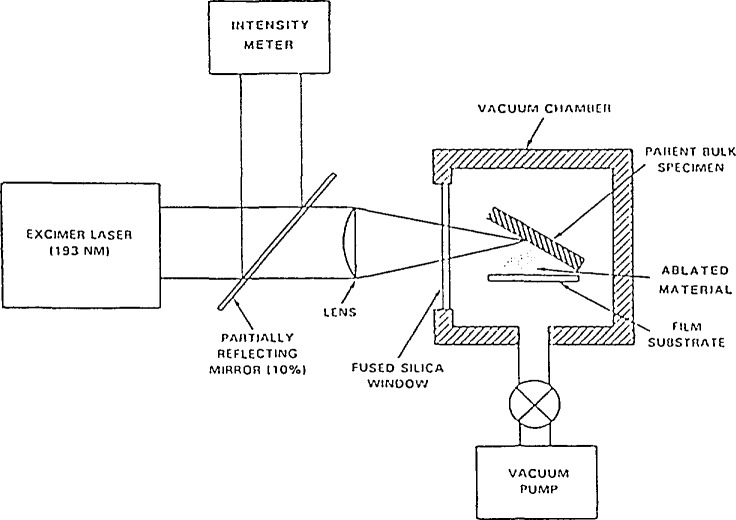
Schematic of laser-ablation process [141].

The making of YBCO films was also investigated using two different techniques. In the first approach, films were made from a bulk superconductor by laser ablation [51], as shown in fig. 8. A pulsed-laser source was used to vaporize the surface of a disk made from superconducting YBCO and deposit a film on a fused silica substrate. Films were made by irradiating a spot or raster scanning. The resulting films were 1 cm^2^ in area and thicker in the center, ~ 2 *μ*m, than on the edges. The as-deposited films had superconducting regions with properties comparable to the bulk material. Similar results were obtained for La-Sr-Cu-O (LSCO). The potential advantage of this method is that the film does not need a high temperature anneal to incorporate oxygen. This is important in hybrid electronic (superconductor-semiconductor) applications where a heat treatment could destroy the semiconductor.

**Figure 9:**
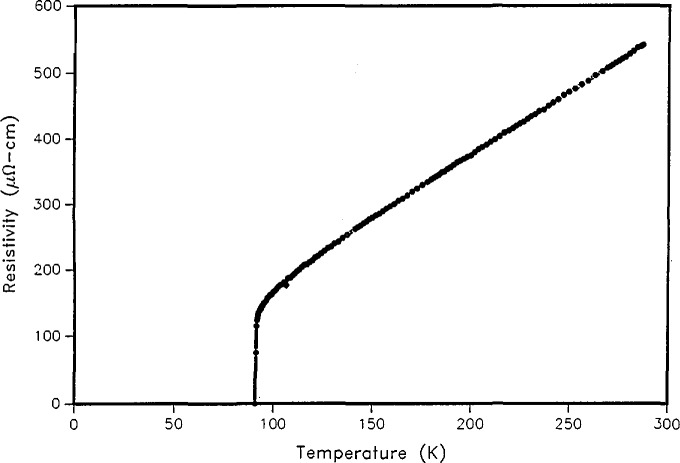
Resistive transition of YBCO film made by coevaporation of Y, Cu, and BaF_2_ on a SrTiO_3_ substrate [52].

A second technique investigated for making YBCO films [52] utilized co-evaporation of Y, Cu, and BaF_2_. These materials were deposited simultaneously onto a room-temperature substrate. Oxygen was introduced into the vacuum system during deposition. At this stage, films containing BaF_2_ are tolerant to moisture, air, positive photoresist, developer, and common solvents. Annealing in oxygen and water vapor incorporated additional oxygen into the film and reacted away the fluorine. The choice of substrate is critical for many applications. The best films have been fabricated on Sr-TiO_3_. A resistive transition, about 0.5 K wide, is shown in fig. 9.

Films deposited using the second method are patterned using conventional photolithographic processes [52]. Prior to deposition of the YBCO, the substrate is coated with photoresist, exposed with a pattern using a projection printer, and developed. The exposed and developed resist exposes bare substrate where the patterned YBCO is to remain. After the room temperature deposition of the Y, Cu, and BaF_2_, but before the oxygen anneal, the remaining photoresist is dissolved, removing the unwanted portions of the film. Annealing in oxygen as before creates a patterned superconducting film.

Superconducting strips with dimensions as small as 1.5 *μ*m have been successfully fabricated. A patterned film having a constriction of 5×5 *μ*m had a critical current density of 5.6×l0^6^A/cm^2^ at 4 K. As mentioned earlier, such high critical current densities are not yet achievable in bulk samples. Patterned films of this sort are being used in fundamental studies of noise in small constrictions and as transition edge bolometers. Efforts to make high-*T*_c_ Josephson junctions are in progress.

## 6. Processing-Property Relationships

**Figure 10:**
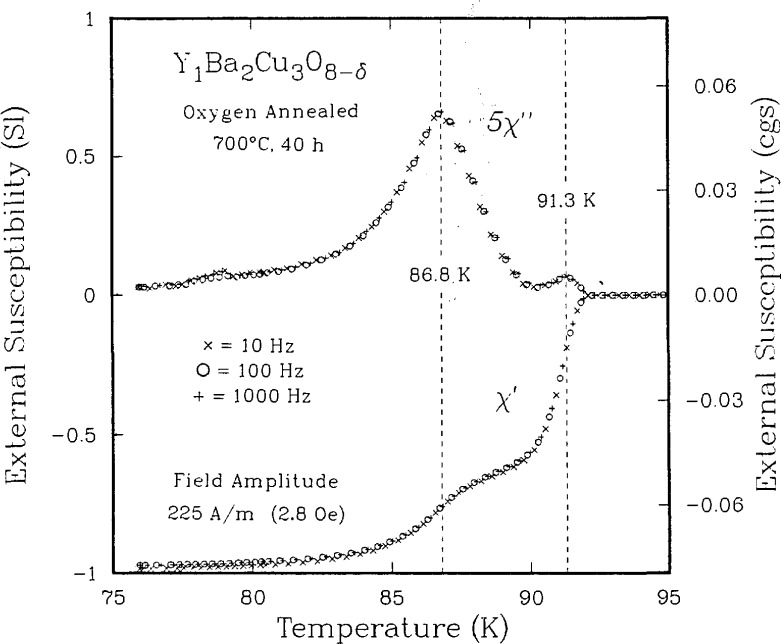
ac susceptibility vs temperature for YBCO. In the imaginary part, two peaks are apparent. Note that the susceptibility is almost independent of frequency [55].

**Figure 11:**
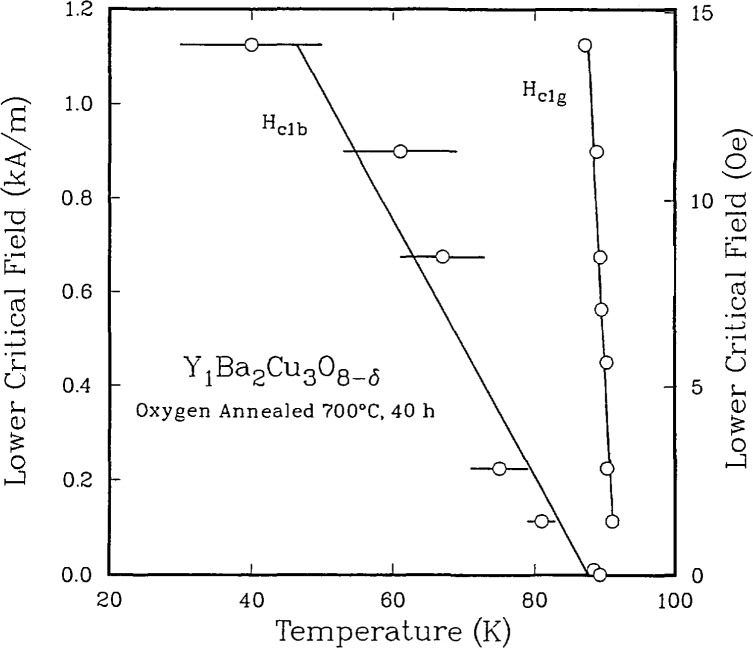
Lower critical fields vs temperature for the two components observed in figure 8. The g refers to the higher *T*_C_ component [55].

The understanding of the relationship between microstructure, processing, and properties is particularly important for the high-temperature superconductors. These are oxygen-sensitive, brittle materials whose processing parameters need to be controlled to produce optimum properties. The understanding of this relationship requires, in addition to the measurement of electrical conductivity, techniques such as x-ray diffraction, magnetic susceptibility, and ultrasonics. Neutron activation analysis can be used to determine the stoichiometry [53]. ac susceptibility measurements can be used to characterize the superconducting properties. The ac data consist of a real and an imaginary component. The real part can be used to determine *T*_C_ and to estimate the percentage of superconducting sample. There have been questions about the interpretation of the imaginary part. Goldfarb et al. [54] have provided evidence that the imaginary part is almost totally due to hysteresis losses and have shown how the temperature at which the slope of the imaginary component becomes positive upon warming can be used to estimate *H*_c1_. To observe a sharp magnetic transition and complete bulk diamagnetism, the applied measuring field must be very small. As shown in figure 10, two distinct superconducting components in a single-phase specimen were identified [55]—one a relatively high *T*_c_, *H*_c1_ superconductor and the other a relatively low *T*_c_, *H*_c1_ superconductor (see fig. 11). These two components were found in all sintered high-temperature superconductors that were examined. The results of subsequent experiments on sintered and powdered samples suggested that the first component was intrinsic to the material, while the second arose from inter-granular coupling [56].

**Figure 12:**
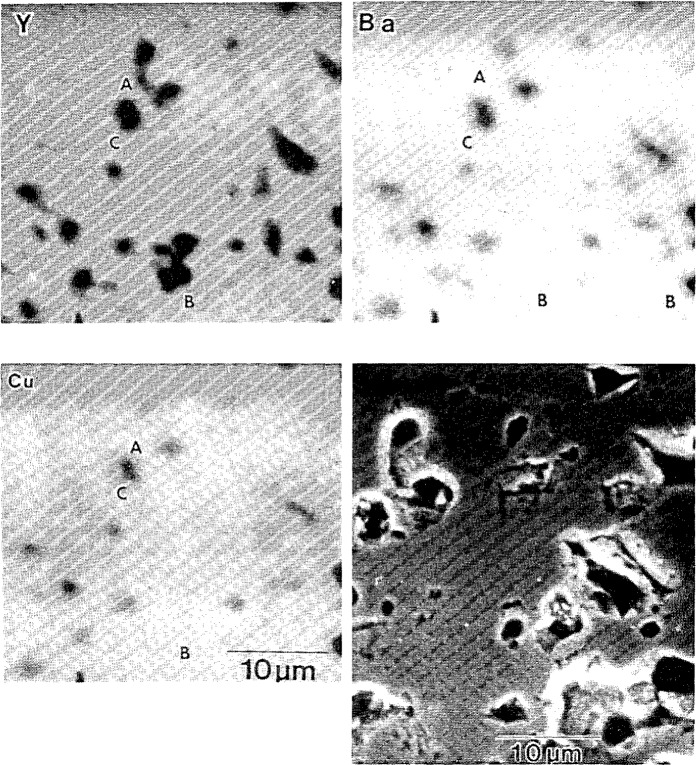
Electron-microprobe compositional maps for Y, Ba, and Cu and corresponding SEM image of YBCO sample. Region A shows a decrease in yttrium concentration, but no copper or barium enhancement; B shows an yttrium-poor region corresponding to a barium-rich but unchanged copper region; C shows an enhancement of barium with no changes in copper or yttrium concentrations [58].

Since the superconducting properties of the new superconductors are strongly dependent on microstructure and composition, techniques available for elemental and molecular microanalysis, principally, electron-probe compositional mapping and micro-Raman spectroscopy were employed to investigate a variety of samples in the YBCO system. Electron-probe compositional mapping is computer-aided x-ray microanalysis furnishing spatially-resolved digital images in which the displayed grey scale is related to the true composition of the specimen and not merely to x-ray intensity of any given element. In studying YBCO, three wavelength dispersive spectrometers were employed; one each for the detection of yttrium, barium, and copper. A representative result is shown in figure 12. Compositional mapping is most useful, therefore, in the identification of compositional heterogeneities on the micrometer-scale, and the determination of dissimilar phases in a high-temperature superconductor [57–59].

**Figure 13:**
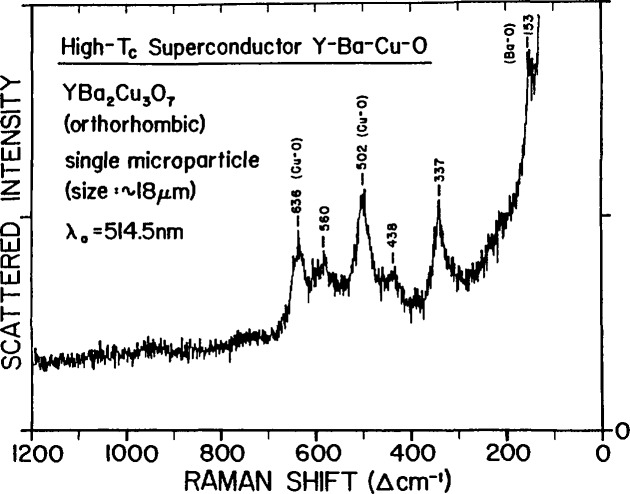
Micro-Raman spectrum of an arbitrary isolated particle of the superconductor YBa_2_Cu_3_O_7−_*_x_* (*x*≈0) identified to be in the orthorhombic phase. The spectrum is excited with the 514.5 nm line of an argon/krypton ion laser at low irradiance, employing 5 mW in a ~ 12 *μ*m beam spot. The microparticle is supported by a lithium fluoride substrate. The resolution is 7 cm^−1^ [60].

Raman and infrared spectroscopy are widely used tools for investigating and characterizing high-*T*_c_ superconductors. The Raman spectra exhibit vibrational modes mostly related to Cu-O bonds and to vibrations of other atoms in the lattice. The spectra are sensitive to differences in crystal structure, bonding, and phase relationship and, furthermore, show a variation with oxygen content, thus providing information on oxygen stoichiometry. Micro-Raman spectroscopy extends these capabilities into the microscopic domain with a spatial resolution comparable to that of electron probe microanalysis. Preliminary work [60] has shown that this technique can provide molecular information not revealed by macro or average structure methods. A micro-Raman spectrum of the YBCO superconducting ceramic in the orthorhombic phase is shown in figure 13. Any variations in the frequency positions and relative intensities of the bands observed in these spectra are indicative of compositional and structural differences attesting to sample heterogeneities.

Lattice defects in YBCO can be identified by field-ion microscopy (FIM). This technique permits the qualitative determination of surface and bulk atomic configurations and microstructural features. Atomic striations observed in FIM images are possibly due to preferentially conducting layers in the material. Thus the superconductivity is possibly localized to specific layers, which are tentatively identified as the Cu-O planes of the orthorhombic unit cell. FIM identified various lattice defects such as dislocations and grain boundaries in the superconductors YbBa_2_Cu_3_O_7_*_−x_*, SmBa_2_Cu_3_O_7_*_−x_*, GdBa_2_(Cu_.96_Fe_.04_)_3_O_7_*_−x_*, and GdBa_2_(Cu_.92_Fe_.08_)O_7_*_−x_* (0<×<.5), in addition to YBCO [61–64].

**Figure 14:**
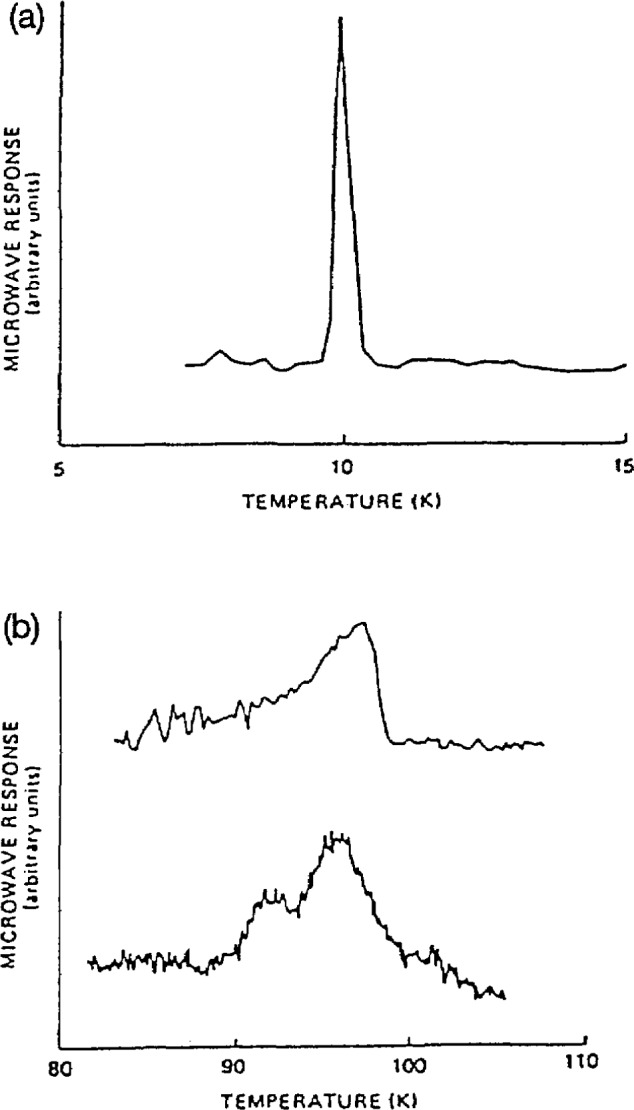
a) Microwave (MAMMA) signal vs temperature for a bulk sample of niobium. The *T*_C_ obtained was in good agreement with known values of *T*_C_ for niobium, b) Microwave (MAMMA) signal vs temperature for bulk YBCO (above) and thin film (below) made by laser ablation from the bulk. The value of *T*_C_=95 K is in good agreement with resistivity and Meissner data. The double peak for the thin film is indicative of two phases with slightly different *T*_C_’s [51].

A new technique has been developed to observe the superconducting transitions—magnetic-field-modulated microwave absorption (MAMMA) detection [51, 65]—which differs from conventional microwave techniques in that it observes only magnetic-field induced changes in the sample’s microwave loss as a function of temperature. The technique is accomplished in a conventional ESR spectrometer by applying a small ac magnetic field to the sample and phase detecting the microwave power reflected from the cavity at the ac modulation frequency. It has the advantages of ease of implementation using commercial ESR apparatus: high sensitivity due to noise reduction by narrowband amplification and phase-sensitive detection, and selectivity since only changes in sensitivity which are magnetic field dependent will be observed. The latter is characteristic of a superconductive transition, as illustrated in figure 14a. This MAMMA technique has been used to study bulk and film specimens of lanthanum, yttrium, and bismuth high-temperature superconductors (fig. 14b). These films were prepared from oxide targets by laser ablation [51].

The composition and microstructure of YBCO was studied as a function of processing [45]. Strontium was found to be the major contaminant. The starting compositions were barium rich relative to the Y:B:Cu ratio, which remained constant during processing. Electron-probe microanalysis revealed three types of inhomogeneities that are within regions which correspond to the YBCO composition—(i) Ba-rich; Y, Cu-poor, (ii) Y-rich, Ba-poor, and (iii) Cu-rich with lesser amounts of Ba and Y. These phases have been identified as (i) BaCu_2_O_4_, (ii) BaY_2_CuO_3_, and (iii) the remnants of a liquid phase that is present at the sintering temperature. The liquid phase limits *J*_c_ since the intergranular phases are not superconducting. Another source of insulating grain boundary film is carbon, which may arise from atmospheric CO_2_ and from solvents used during grinding. During low-temperature oxidation of the sintered material, residual carbon may react with oxygen to form gas-filled pores along the grain boundary and a high concentration of defects adjacent to the grain boundary. Hence, it is apparent that a large degree of compositional control is needed to control the properties of YBCO.

Processing-property relationships for YBCO have been studied as a function of annealing temperature and environment. It was found [57, 58, 66] that annealing at low temperature in oxygen is necessary to obtain the highest *T*_c_, sharpest transition, and the largest superconducting fraction. Samples contained a small amount of inhomogeneous second phase liquid, insufficient to prevent current flow. Sintering was more rapid and to a higher density in air than in oxygen. Segregation occurred during sintering and pores and micro-cracks were observed. The thermal expansion was very high for a ceramic—indicating that thermal shock may be a problem for these materials. Fracture toughness was quite low and the material was susceptible to moisture-enhanced cracking.

**Figure 15:**
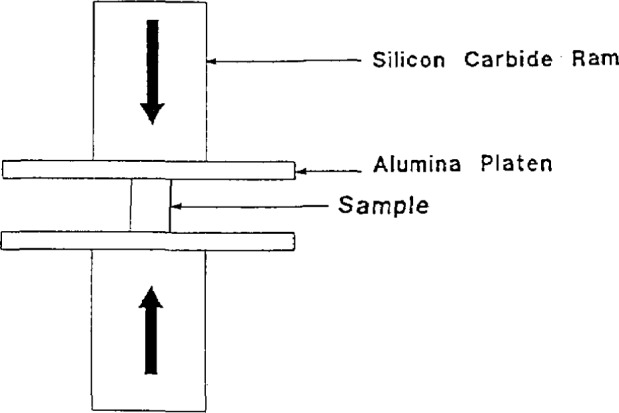
Sinter-forging apparatus. The load was applied in the vertical direction with no die wall constraints. The sample was separated from the ram by alumina plates [67].

Sintering at 950 °C gave the best density but poorest superconductivity due to a lack of porosity required for oxygen diffusion. Sinter-forging was investigated [66] reasoning that it should be possible to increase the current density by aligning the grains. The grains had a high density center with the edges cracked and not very dense. Large yttrium-rich bands were formed perpendicular to the stress direction as a result of local segregation. The apparatus is shown in figure 15. The *c* axis tended to be aligned parallel to the applied stress direction. The transport *J*_c_ was less than 100 A/cm^2^, however, and in some cases even zero. This was due to weak linking. Transmission electron microscopy showed second phases at grain boundaries, forming S-N-S junctions [67].

**Figure 16:**
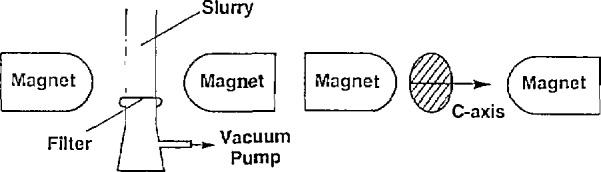
Configuration for casting in a magnetic field. The slurry of isoproponal and powder was placed in a homogeneous 2 T magnetic field [68].

Another possible method to align the grains would be to cast the samples in a magnetic field. The alignment is due to anisotropy of the paramagnetic susceptibility of the grains. Ostertag et al. [68] studied the magnetic casting of YBCO and HBCO (H=holmium). A slurry of the superconducting powder and isopropanol was placed in a homogeneous magnetic field of 2 T for 30 minutes (see fig. 16). The samples, which were then pressed and sintered, tended to align with their *c* axes parallel to the applied field ([001] alignment). However, this alignment is not sufficient for high *J*_c_ Alignment in the [010] and [100] or [010] is also needed since tilt decreases *J*_c_ Clean grain boundaries are also a requirement. Current densities of samples aligned in the oxygen-rich state were up to five times greater than samples aligned in an oxygen-deficient state and then oxygenated, due to the presence of non-superconducting junctions. Bulk *J*_c_ calculated from magnetic measurements were 10^3^–10^4^ A/cm^2^.

## 7. Electrical Contacts

One problem that existed in the study of high-temperature superconductors was too high a resistivity in the electrical contacts. Contacts made of indium solder, silver paint or epoxy, direct wire bonds, and pressure contacts have contact surface resistivities in the range of 10^−2^ to 10 Ω-cm^2^ which is several orders of magnitude too high for measurement and applications. Contact resistivities of 10^−4^ to 10^−5^ Ω-cm^2^ or lower are needed. Ekin and coworkers [69–71] developed a method consisting of sputter etching the surface of the superconductor to remove the degraded surface area immediately before depositing noble metal (Ag or Au) pads, followed by annealing the noble metal/superconductor interface in oxygen. Contact resistance for the silver pads showed metallic behavior, decreasing by a factor of 3 to 12 as the temperature decreased from 295 to 76 K. Contact surface resistivities less than 10 *μ*Ω cm^2^ at 76 K were achieved without oxygen annealing. After annealing in oxygen at 500 °C for 1 hour, contact resistivities were reduced to as low as 0.1 nΩ cm^2^ [70]. The low oxygen affinity of the noble metals may play an important role in passivating the contact interface. On the other hand, oxygen and indium formed a semiconducting oxide with resistivities greater than that of pure indium [70, 72]. Room-temperature diffusion of oxygen is limited in the noble metals, thus protecting the YBCO. This may explain why there can be low contact resistance despite exposure of the YBCO to air.

Moreland and Goodrich [73] have developed silver screen contacts for rapid characterization of YBCO. The screens can be used for making voltage contacts and voltage taps. Silver wire screens are interleaved between calcined powder sections and fixed to form a composite pellet. Silver diffuses in the powder during sintering to form proximity contacts permeable to oxygen.

## 8. Electronic Structure

**Figure 17:**
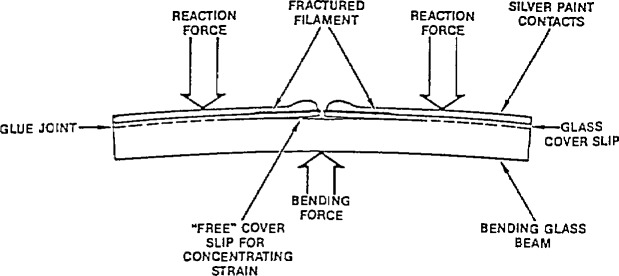
Fabricating a break junction. A superconducting filament is mounted on a beam which is bent using an electromagnetic force. Once the filament is fractured, the beam is relaxed to form a tunneling contact within the fracture of the filament. Contact may be either through a thin insulating medium (vacuum, gas or liquid) or by closing the fracture to form a point contact. An electromagnetic assembly affords precise control of the tunneling gap [84].

**Figure 18:**
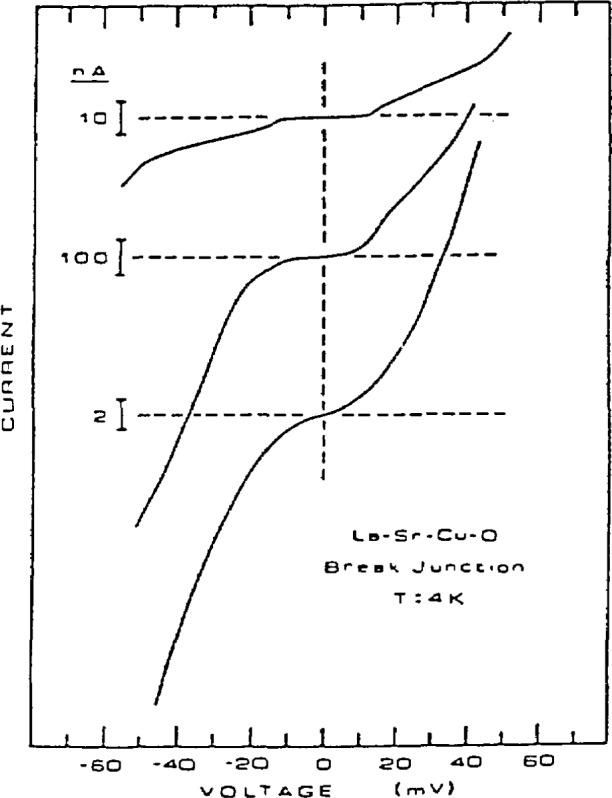
Voltage vs current curves for a LSCO electron tunneling break junction immersed in liquid helium at 4 K for three different barrier settings. The bottom curve was the most common V-I characteristic found [75].

One method of obtaining information on the electronic structure of superconductors is by tunneling measurements. A technique used for such measurements was developed by Moreland and Ekin [74]—the break-junction technique (fig. 17). In a break junction, tunneling occurs across the fracture of a bulk sample. A small piece of a bulk material is mechanically fractured under liquid helium and the freshly fractured surfaces are adjusted to form a tunneling barrier with helium as the insulator. The sample can be a single crystal, polycrystal, or sintered pellet. Unlike other tunneling techniques, break junctions give information on the interior of bulk samples. Break junctions have been used to study both the lanthanum and yttrium superconductors. Tunneling junctions for LSCO (fig. 18) exhibited a variety of tunneling behavior [75–77]. Scanning-electron microscopy showed a rough surface with numerous voids and scattered inclusions. This variability may be due to tunneling between different phases in the material. Large energy gaps and deep structure in the conductance derivatives are evidence for a strong coupling mechanism.

Break junctions for YBCO gave results indicative of strongly coupled superconductors [78] but had the same variability as LSCO. Variable results in perovskites can be explained as being due to the structure which consists of alternating layers of insulating and conducting platelets which can be superconducting, semiconducting, or both [77]. While evidence for the usual pairing state associated with the BCS theory was found, so was a lower *I*_c_R product which is indicative of a lower energy gap than that expected from BCS theory. In addition, a Josephson junction effect was found [79]. Evidence of an intrinsic energy gap was found in both LSCO and YBCO [80]. The gap scales with *T*_c_ and decreases and vanishes when approaching *T*_c_ from a lower temperature. This points to the energy gap being quasiparticle in nature.

Break junctions in single crystals should permit a direct measurement of gap anisotropy if the samples are fractured along cleavage planes. To this end, break junctions of single crystal HoBa_2_Cu_3_O*_x_* (HBCO) were compared with polycrystalline YBCO [81]. Both had junction conductance increasing linearly with junction bias. Gap structure of YBCO occurred more often during adjustment of the junctions than with HBCO. This may have been due to a lack of oxygen penetration in the single crystal. The results may have been affected by the fact that the HBCO fracture surfaces were not ideal. The V-I curves showed the square-law dependence of current seen in many tunneling measurements of polycrystalline YBCO.

Based on the fact that the anomalies in the break junction results may be microstructural in origin and not due to the electron coupling mechanisms, several models have been proposed. In the granular model [82], the superconductor is divided into grains isolated from each other by insulating tunneling junctions. A second model, the multiparticle model [83], assumes that the grains are oriented to form a series array of junctions near a primary tunneling contact. Moreland et al. have developed a third model to explain these results in perovskites [84]. In the laminar model, the microstructure consists of a complex tunneling matrix with parallel superconducting laminae connected to each other, the point contact, and the surrounding grains by tunneling junctions. This structure may be manifested in a layered perovskite single grain with superconducting layers separated by high dielectric insulating barriers. The individual laminae form a series-parallel network of superconducting junctions within a single grain of the material. Although there is some evidence that casts doubt upon the granular model, the exact model is still in question.

Tunneling measurements were also made on YBCO thin films [85] using the method of squeezable electron tunneling (SET) junctions developed by Moreland et al. [86]. In contrast to the break junction measurements of bulk samples where the spectra are often without energy gap features, SET spectra invariably contain such features. This implies that the film is superconducting near the surface, in contrast to results on bulk materials which indicate that only parts of the interior are superconducting. Improvement of surfaces by the addition of very thin noble metal films, which become superconducting by the proximity effect, is under investigation [87].

**Figure 19:**
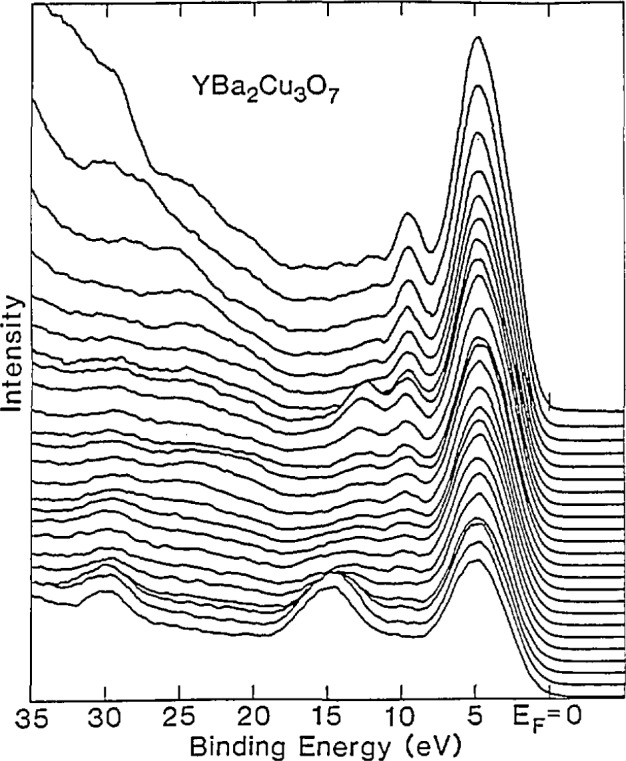
Ultraviolet photoemission spectra of fractured YBCO. The top curve is at h=60 eV, the bottom 106 eV, and each curve is separated by 2 eV. At 60 eV there are two valence band features—at binding energies of 5 and 9.4 eV. Increasing the photon energy, features become apparent at 12.4, 15,0, and 28.8 eV. The features at 9.4 eV are due to Y/Cu; at 12.4 eV to Cu, and at 15.0 and 28.8 eV, Ba [89].

Measurement of the electronic structures of the high-temperature superconductors are important in providing supporting evidence for theoretical models of superconductivity. Kurtz [88] has written a review of the experimental measurements of the valence electronic structure of LSCO and YBCO. NIST’s Synchrotron Ultraviolet Radiation Facility (SURF-II) was used to study these features. The electron structure of YBCO was measured using resonant photoemission, which is associated with the enhancement of valence photoelectron features resulting from the coupling of excitation and decay mechanisms at the core-electron photoabsorption onsets. Radiation in the 60-160 eV range was used [89], (fig. 19). The upper edge of the valence band was found to nearly coincide with the Fermi level and the density of states was small. There was no distinctive edge. The valence band did not resonate with the photon energy. Furthermore, there was no evidence of valence band structural changes as the temperature was lowered below the critical temperature. The copper oxide in the material was found to give spectra similar to CuO.

Another study using SURF-II but at an energy range of 20–600 eV [90], confirmed the 2^+^ valency of copper in YBCO. The National Synchrotron Light Source at Brookhaven was used to provide information on oxygen, barium, and yttrium. It was found that the p-type partial density of states is very small at the Fermi energy. The electronic structure observed in the photoemission measurements is associated with the oxygen 2*p* orbitals. This study also observed no change in the spectra as the temperature was lowered below the critical temperature.

Additional studies carried out by Kurtz, Stockbauer, and coworkers included photoemission of YBCO and LSCO, which revealed a resonance in the peak located at a binding energy of ~9.5 eV for photon energies spanning the onset of O–2*s* excitations. This feature is associated with oxygen excitations. The satellite is suppressed on surfaces that are superconducting within the probe depth of the spectroscopy [91]. Photoelectron spectroscopy of high-*T*_c_ superconductors, including the newer bismuth and thallium superconductors revealed that the materials have a highly hybridized Cu-O valence band and resonant satellites which imply that the materials are highly connected. No substantial changes were observed in the electronic structure as the materials were cooled from room temperature to below *T*_c_. The materials reacted strongly with H_2_O and CO_2_, forming hydroxides and carbonates, but reacted more weakly with O_2_ and CO [92, 93].

Photoemission measurements of YBCO revealed two constraints on any theoretical treatments of its electronic structure based on the observation of a 2.3 eV feature [94]. First, YBCO has a higher charge carrier concentration at the Fermi level than in related lower-*T*_c_ and non-superconducting compounds. Secondly, there is a large contribution from oxygen to the density of states near the Fermi level, mainly derived from oxygen in the Cu-O chains. The 2.3 eV feature is intense in the orthorhombic phase, but weak in the tetragonal.

## 9. Physical Properties

**Figure 20:**
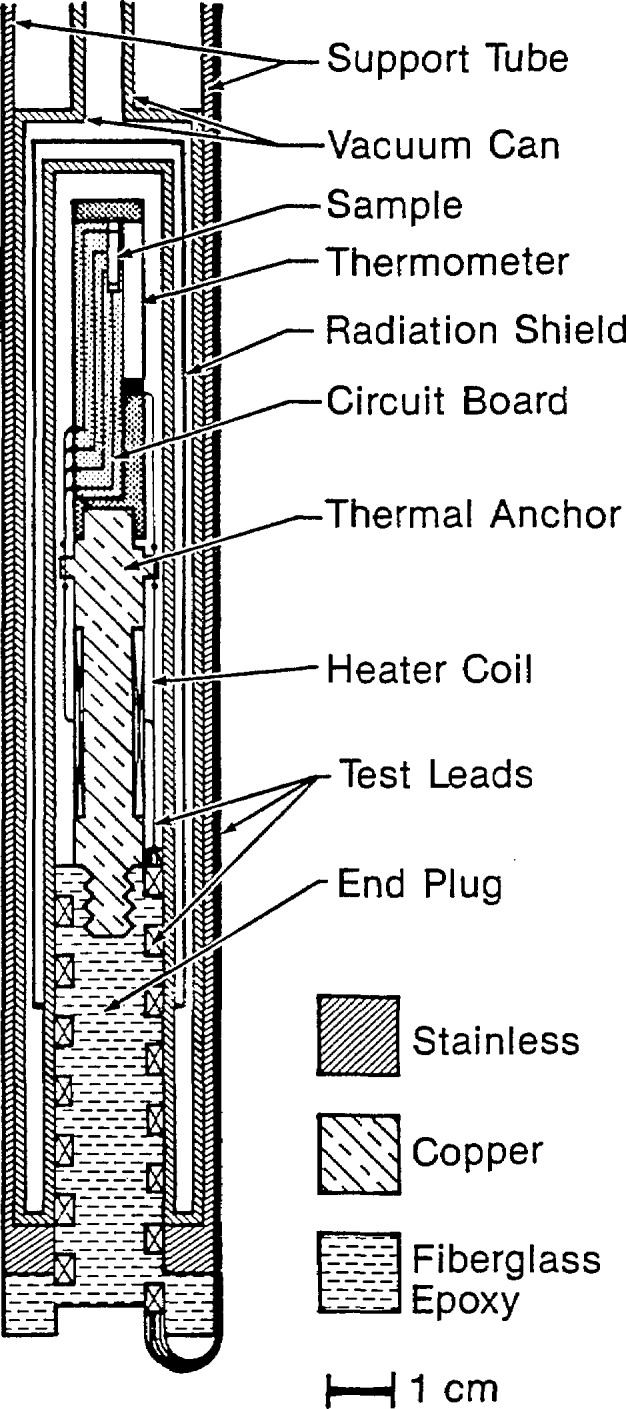
Cryogenic bathysphere for resistance measurements of high-*T*_C_ superconductors [95, 96].

Current densities are a critical parameter for the successful application of high-temperature superconductors. A cryogenic bathysphere developed by Moreland et al. [95,96] for resistance measurements of high-*T*_c_ superconductors is shown in figure 20. This device thermally isolates an environmental chamber from surrounding cryogenic fluids. The bathysphere has the advantages of (i) being compact enough to fit in the base of a high-field superconducting solenoid without the use of a re-entrant dewar; (ii) the sample remaining dry; (iii) being inexpensive; (iv) having no moving parts; and (v) having sufficient thermal contact between sample and thermometer provided by the ambient pressure exchange gas to maintain thermal equilibrium within ±0.1 K while the temperature changes as fast as 3 K/min. It may also be possible to adapt this device to susceptibility, critical current, and electron tunneling measurements. The bathysphere has been successfully tested with NbTi in liquid helium and YBCO in liquid nitrogen.

**Figure 21:**
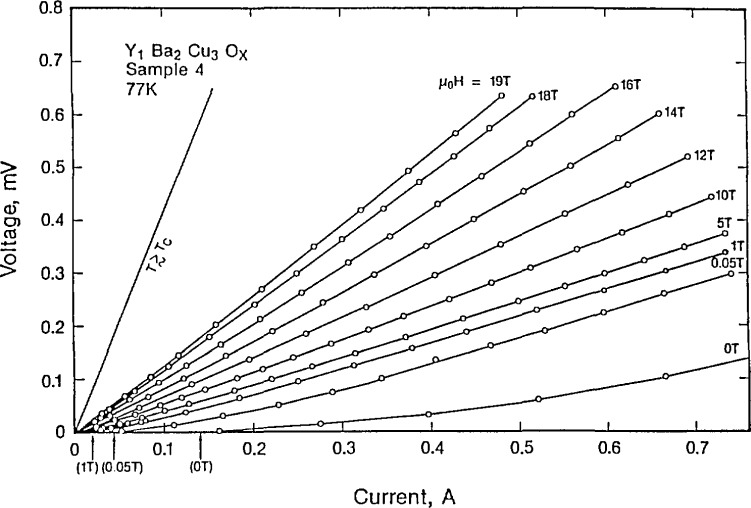
Voltage vs current characteristics for a YBCO sample in transverse magnetic fields in liquid nitrogen at 77 K. High magnetic fields were required to increase the slope to the normal resistance value at *T*_C_, and the transport critical current is suppressed by very low fields. The curves are nearly linear at currents well above the critical currents indicated by the arrows [100].

As previously mentioned, current densities of the order of one million A/cm^2^ will be required for most applications. While films with these current densities have been produced, bulk materials have had much lower current densities. Ekin et al. [97, 98] studied bulk sintered YBCO samples from several different laboratories. Using V-I characteristics, they found that while a field of over 30 *T* was needed to suppress all superconductivity, a field of only a few tesla could suppress the transport current (fig. 21). The measured transport current was significantly lower than that measured by magnetization. The superconducting transition in polycrystalline YBCO is very broad. This is consistent with a model of a weak-link region between high-current-density grains. At least part of the behavior is due to intrinsic conduction anisotropy. This an-isotropy has been observed in YBCO single crystals with the weakest conduction along the *c* axis [99]. The low current density could be due to: (i) impurities or low-*T*_C_ phases at the grain boundaries or (ii) misalignment of the grains. Electron microscopy gives no evidence of the former [100]. The location of the weak links in YBCO could be at the grain boundaries, within the grains or between the Cu-O planes. Transport critical current densities have been measured at low magnetic fields in several kinds of high-*T*_c_ superconductors fabricated in many different laboratories, and fitted with a model which assumes that the barriers to current flow are Josephson weak links which have a statistical distribution of sizes and orientations [101–103]. The data were shown to follow the Airy current-field pattern. The fits of the data to theory are good for all the samples. The fitting parameter essentially gives the average dimension of the junctions, which in all instances is about equal to the grain size, thus furnishing convincing evidence that the barriers at low magnetic fields are at the grain boundaries. This finding indicates that a possible method for increasing the current density would be by processing in such a manner that the grains would be aligned.

Other physical properties of interest include the elastic constants, which have practical significance, such as in stress and fracture toughness, and are related to physical properties such as specific heat and hardness. Elastic constants relate strongly to interatomic potentials and force constants and can be used to calculate the Debye temperature which is used in the BCS calculation of the critical temperature. They also relate strongly to any phonon-mediated superconductivity mechanism. Values of elastic constants can be determined by ultrasonic methods and one of them, the bulk modulus, by x-ray diffraction. The elastic properties of metal-oxide superconductors have been reviewed by Ledbetter [104].

**Figure 22:**
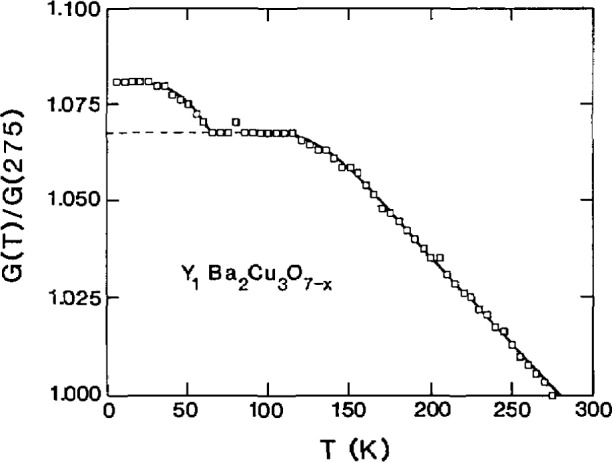
Relative shear modulus *G=pv_t_*^2^ between 275 and 4 K for YBCO. Above *T*_C_ (65 K for this material), behavior is normal. Below *T*_C_, contrary to expectation, *G* apparently increases. However, a reentrant-softening model reconciles this apparent anomaly [107].

Ledbetter and coworkers have measured the elastic constants of YBCO using ultrasonic techniques. YBCO was compared to BaTiO_3_ [105] and was found to have a lower elastic stiffness which could arise from oxygen vacancies or microcracks. The latter have a larger effect than a comparable fraction of spherical voids. Study of six YBCO specimens [106] showed that some specimens may be free from softening defects and their properties may reflect intrinsic behavior. A check on this is to compare elastic and thermal Debye characteristic temperatures. Elastic constants were measured as the specimens were cooled through the transition temperature [107–110]. Samples run in helium gave reproducible results suggesting that these measurements represented intrinsic material properties. Elastic constants showed irregularities above and below, but not at, the critical temperature. The shear-modulus results (fig. 22) departed from those expected for a simple second-order normal/superconducting transition, in agreement with the results for the dilation [111]. The value of the Poisson ratio behaved irregularly below the transition temperature indicating a change in interatomic forces supporting Geballe’s view [87, 112] that a large fraction of electrons enter into Cooper pairs, the gap is approximately equal to the Fermi energy, and coupling is strong. During cooling from 160–70 K, YBCO behaved as if it underwent a sluggish phase transition. Two YBCO materials with different oxygen contents, *x* =6.70 and 6.92, showed similar ambient-temperature elastic-constant values, and similar temperature behavior [113], but the *x* =6.92 YBCO demonstrated a higher elastic stiffening during cooling to 4 K.

The behavior of the elastic constants can be described by a “reentrant softening” model [114, 115]. Softening occurs just above the critical temperature suggesting growing lattice instability with decreasing temperature. Premonitory behavior of this type is known to be associated with martensitic or displacive structural transformations in various materials including the A15 superconductors [116]. The increased stiffness below the transition temperature is the result of the softening being offset by the increased stiffness associated with the developing superconducting phase. The calculation of the Debye temperature based on this model is in agreement with other experimental measurements. The model also predicts that the elastic constants will have a higher value in the normal state than in the superconducting state. The results of measurements on LSCO are also in agreement with this model. Strong thermal hysteresis, especially in the dilational modes, were found in subsequent studies by Ledbetter and Kim [117].

**Figure 23:**
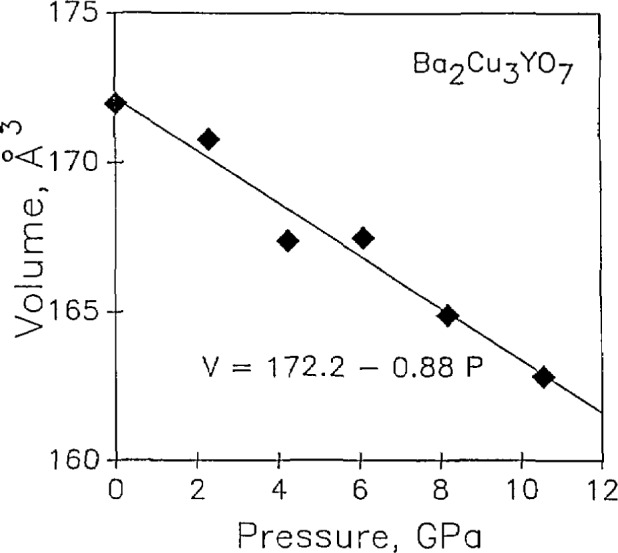
The pressure dependence of the volume of YBCO. The lattice parameters are determined from x-ray diffraction data. These data are used to calculate the isothermal bulk modulus [118].

The bulk modulus of YBCO was determined by measurements in a diamond-anvil cell using an energy dispersive x-ray diffraction technique [118]. The least compression was observed within the perovskite layers because of the oxygen packing, and the largest was observed perpendicular to these layers. As seen in figure 23, the decrease in volume was essentially linear with applied pressure. The value of the bulk modulus was larger than that determined by ultrasonic techniques. Ledbetter and Lei [119] focused on this difference and its implication for the related Grüneisen parameter, supporting their measurements by ionic-bonding calculations.

Ultrasonics can also be used to provide additional information [120]. The ultrasonic velocity in YBCO was found to be different on warming than on cooling, with the greatest difference occurring in the first cycle. Three attenuation peaks were found on warming: I at 65–75 K, II at 134 K and III at 183 K. The hysteretic velocity changes and peak I appear related to a first-order phase transition involving magnetic superstructure in non-superconducting portions of the sample. Peak III appears to be consistent with a defect relaxation process. The origin of peak II, which was dependent on thermal history, could not be identified.

**Figure 24:**
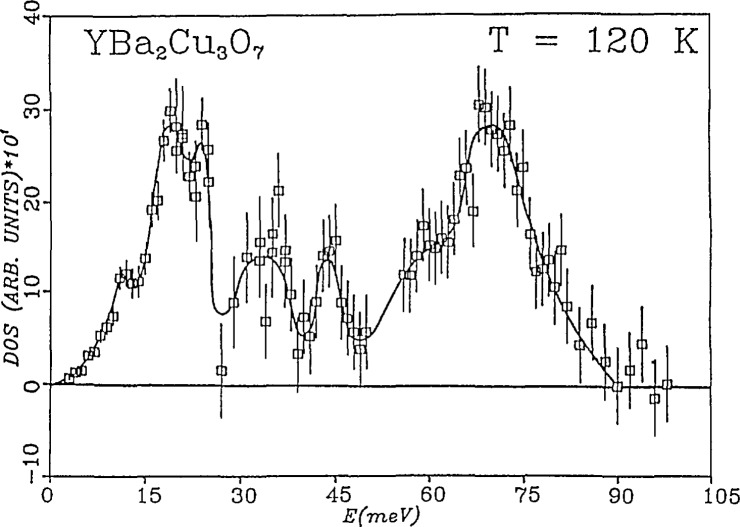
Vibrational density of states as measured with inelastic neutron scattering at 120 K. The largest spectral weight is contained in peaks involving oxygen vibrations [21].

Neutron inelastic scattering was used to measure the phonon density of states in an attempt to ascertain if any significant perturbations occurred in the phonon modes at the superconducting transition temperature [21]. A “softening” of such modes is a key aspect of conventional phonon-driven superconductivity. The normalized density of states at 120 K for YBa_2_Cu_3_O_7_ is shown in figure 24. The spectra consist of a strong double peak near 20 meV and a second principal maximum at approximately 70 meV. Measurements below the transition temperature gave only negligible changes in the observed spectra which could be accounted for by anharmomic effects, and did not indicate any major changes in the overall phonon modes accompanying the superconducting transition. Oxygen-deficient YBa_2_Cu_3_O_7_*_−x_* showed a pronounced weakening of the 70 meV features in the density of states and a filling in and broadening of the lower energy features, reflecting a change in the harmonic modes associated with the absence of oxygen in the Cu-O “chain” structure. These features are equivalent to modes observed by Raman scattering [60].

**Figure 25:**
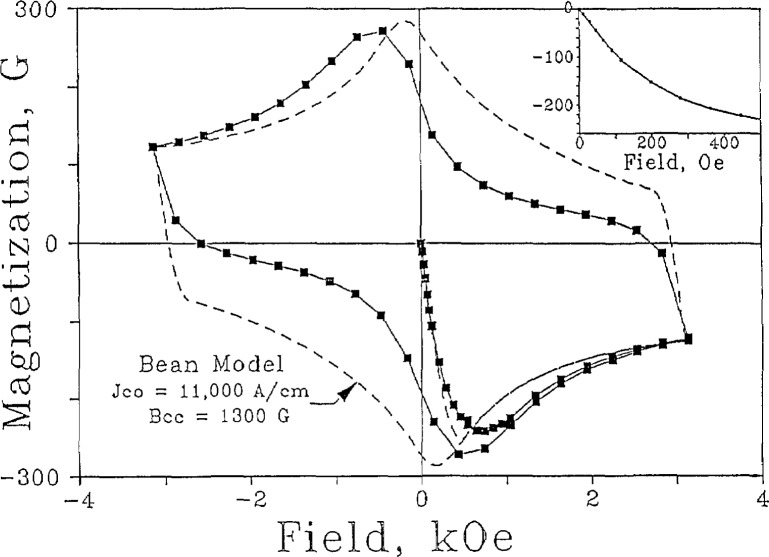
Experimental (solid line) and calculated (dotted line) hysteresis loops for YBCO at 38 K. The insert is an expanded view of the virgin curve [121].

The magnetic hysteresis loops of YBCO were also studied [121]. The shape of the loops well below *T*_c_ (fig. 25) brought to mind the constricted hysteresis loops observed in certain ferromagnetic materials which are usually associated with magnetic aftereffects. Similar dynamic effects with time constants on the order of 10 seconds at 40 K were found to be present in YBCO. This is in addition to flux creep (due to thermally activated jumping over flux-pinning sites) observed for longer time periods. When the measurement time is fast compared to both time constants, the hysteresis loops can be approximated by a critical-state (i.e., Bean-Kim) model [122–126]. The experimental hysteresis loops at higher temperatures are more pinched than the critical-state model because of the movement of fluxoids.

The critical-state model, which provides a method for calculating the energy losses in type II superconductors, has been extended by Peterson [127] to include the train of magnetization jumps often seen at low temperatures in moderate-to-high magnetic fields. Chen and Goldfarb [128] have developed an analytic method for using the critical-state model to determine critical currents from magnetization measurements on the sample shapes most often encountered in developmental studies.

## 10. Theory

The interaction between two test charges in a solid can be described in terms of a total dielectric function that includes electronic and lattice polarization. Stability requirements place restrictions on the dielectric function. Allen et al. [129] show that the eigenvalues of the inverse dielectric matrix, λ_i_, satisfy λ_i_<l. As a result, the electron-electron interaction (as determined by test charges) which enters BCS theory is not restricted to positive values by general stability requirements. Casella [130] considered other intermediate bosons, besides phonons, mediating the superconducting interaction and carried out a semiphenomenological analysis of the effects of certain band-gap features on the gap ratios of high-temperature superconductors. Comparison with experiment suggests that the intermediate boson is not a phonon.

**Figure 26:**
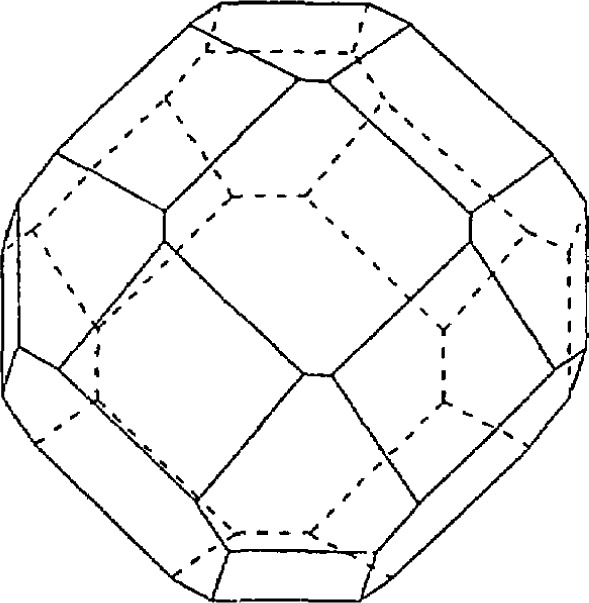
The (0 12 0 8 0 6) Wigner-Seitz polyhedron (coordination number=26) of the Ba, La or Y atom in the ideal ABO_3_ perovskite structure, obtained with the use of metallic radii. In the high-temperature superconductors with distorted perovskite structure, the Wigner-Seitz cells for these sites are derivations from the ideal polyhedron [131].

Melamud et al. [131] studied the near-neighbor environments and the bonding of atoms in lanthanum and yttrium based copper-oxide superconductors using Wigner-Seitz cell construction. Wigner-Seitz cells can identify the nearest neighbors, the site symmetry arising from the presence of these neighbors, and the number of nearest neighbors common to a near-neighbor pair. Different results were obtained depending on whether ionic or covalent/metallic bonding is assumed. Covalent/metallic bonding gave more reasonable chemical results and was consistent with known properties of these materials. The barium, lanthanum, and yttrium atoms all had large coordination numbers (see fig. 26) implying a three-dimensional chemical bonding scheme. The results are in agreement with the conclusion of Pauling [132] that the bonding at the important copper sites is not limited to oxygen but involves substantial interactions with large atoms such as lanthanum and barium.

## 11. Applications

**Figure 27:**
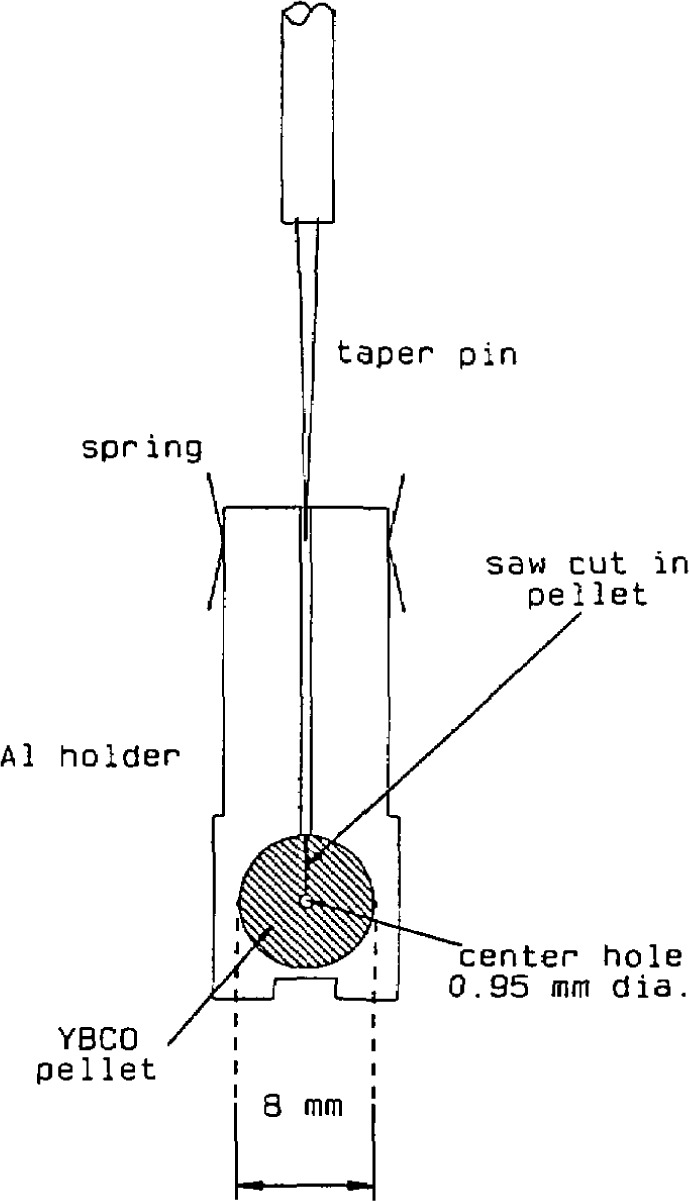
A SQUID made from a YBCO break junction. A YBCO pellet was secured with epoxy in an aluminum breaking fixture. A hole was drilled through the pellet and a saw cut made part way through so that the pellet would break along a diameter when the two arms of the fixture were spread apart by a thin tapered pin. Springs close the break as the taper is withdrawn [133].

Problems with current density and fabrication have hindered many applications of high-temperature superconductors. However, a successful prototype transition edge bolometer and a SQUID (*s*uperconducting *qu*antum *i*nterference *d*evice) made from YBCO have been developed [133, 134]. The breaking fixture used to form the Josephson contact for the SQUID is shown in figure 27. Variations in performance were found with different YBCO batches, and the first devices constructed showed considerable noise above 61 K, although quantum interference effects persisted up to 81 K. However, SQUIDs made from well-characterized, high-quality YBCO, operated in liquid nitrogen, with only a modest increase in noise over that found at 4 K. This provided the first demonstration that sensitive high-*T*_c_ SQUIDs operating at liquid nitrogen temperature are possible.

Many applications for high-temperature superconductivity depend on understanding and improving the critical current. To this end, a YBCO macrobridge (bridge dimensions are much greater than the coherence length) was fabricated [135] to understand not only *J*_c_, but intra-film Josephson effects. Extremely noisy sections of the V-I curve were observed, always well below *T*_c_. This behavior could have ramifications for potential low-noise applications of high-*T*_c_ superconductors. The noise depends on temperature, bias current, and the magnetic field. A very rapid change of switching rate with very small fields and small changes in bias current was observed, which suggests that the noise may be due to the motion of vortices in and out of pinning sites.

The ability of a superconductor to levitate a magnet *above* its surface is well known, and for high-T*_c_* superconductors it is often demonstrated. Recently, it has been realized [136, 137] that specially processed samples of a high-*T*_c_ superconductor can be levitated *below* a magnet. This unusual type of levitation involves “attraction” of the superconductor by a magnet rather than the Meissner effect “repulsion” seen for a levitated magnet. An important application for this effect would be in magnetic bearings (see table 1).

## 12. Other High-temperature Superconductors

Following the discovery of high-temperature superconductivity in Bi-Sr-Ca-Cu-O ceramics [138], Bi_2_Sr_2_CaCu_2_O*_x_* was synthesized both chemically and by a solid state reaction [72]. ac susceptibility measurements showed transitions at 80 K and 110 K and a low *H*_cl_. The appearance and amount of the 110 K superconductor was sensitive to the annealing procedure. Magnetic hysteresis loops constructed at 80 K were narrow, signifying a small amount of trapped flux. The loops were constricted in the center, indicating the probable existence of time effects similar to those seen in YBCO [121]. The bismuth superconductor was also studied by the magnetic-field-modulated-microwave-absorption (MAMMA) technique [65, 139]. Superconducting transitions were observed at 72, 100, and 110K. An applied magnetic field broadened the microwave response peak much more than in the case of YBCO. Thin films of the bismuth superconductor were made [140] by laser ablation on ZrO_2_ and characterized by MAMMA. The film quality was affected by substrate temperature and an annealing process. Unlike previous work [141], the films were not superconducting as deposited.

A classical test to determine the contribution of an electron-phonon interaction to the superconductivity is to measure the isotope shift [3, 4] in *T*_c_. Substitution of ^18^O for ^16^O in the Bi-Sr-Ca-Cu-O system [142], the La-Sr-Cu-0 system [143, 144], and the Y-Ba-Cu-O system [145, 146] has demonstrated a measurable, albeit small, isotope shift in *T*_c_. Although this small effect indicates that the electron-phonon interaction contributes to the superconductivity, it is probably too small to account for the high values of *T*_c_, and other mechanisms, e.g., spin fluctuations must be operative. The possible role of various magnetic interactions have recently been addressed at a workshop held at NIST, Gaithersburg [147].

**Figure 28:**
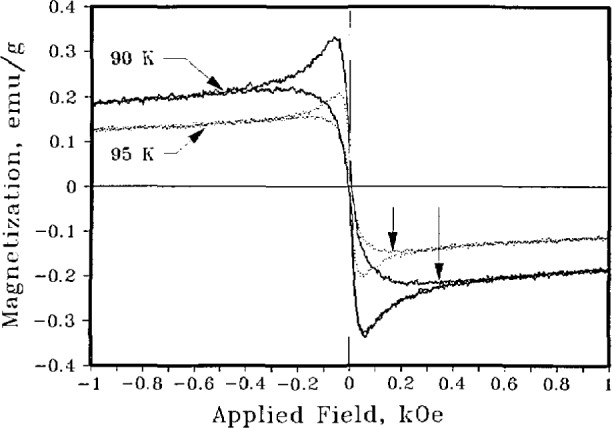
Hysteresis loops at two temperatures for a Bi-Pb-Sr-Ca-Cu-O superconductor, illustrating flux depinning [148].

**Figure 29:**
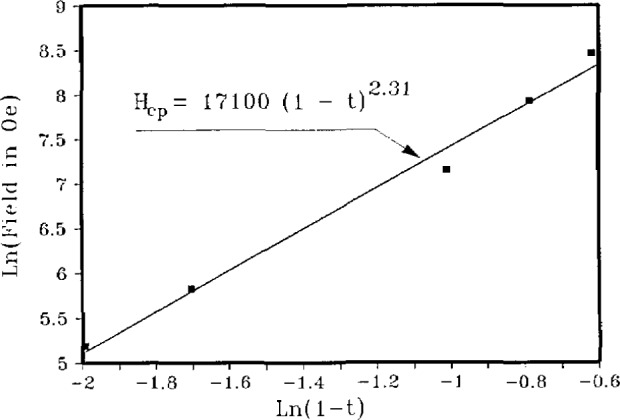
The flux-depinning field of the sample of figure 28 as a function of temperature [149].

Magnetic measurements were made [148, 149] on chemically synthesized Bi-Pb-Sr-Ca-Cu-O. The lead substitution appears to encourage or stabilize the high-*T*_c_ Bi phase. The superconductor displayed extremely narrow hysteresis loops above liquid nitrogen temperatures, indicating a small number of effective flux pinning sites. Below 40 K, a dimpling was observed but only when the sample was a loosely packed powder. A flux depinning was observed, as illustrated in figure 28, for two temperatures. A plot of the flux-depinning field vs temperature appears to be linear (fig. 29).

Ultrasonic elastic-constant studies were carried out for Bi-Pb-Sr-Ca-Cu-O [150], with results similar to YBCO. There was stiffening during cooling, no measurable change at *T*_c_, and hysteresis. However, the Bi-Cu-O is much softer than YBCO, with an elastic Debye temperature of 312 K vs 437 K.

Resonant photoemission has been used to study [151] the electronic states and electron-electron interactions in a bulk sample of Tl-Ba-Ca-Cu-O. The electron structure is similar to that of YBCO indicating that the electron states and interactions are similar. The surface of the Tl superconductor is not as reactive toward atmospheric gases as YBCO.

## 13. Low-Temperature Superconductors

Despite all the current interest in high-temperature superconductors, low-temperature superconductors will still be required in many applications. For example, the cost savings realized by switching from helium to nitrogen for cooling large magnets may be only a small part of the total operating cost. Additionally, much experience has been gained in learning how to fabricate these materials into practical conductors—in the shape of tapes or wires—that can support high current densities under realistic operating conditions. Therefore research on these materials is continuing, with the goal of optimizing their properties—e.g., current-carrying capabilities stability, ac losses, etc.

NIST is developing facilities and standards for the definition and measurement of superconductivity parameters [152, 153]. The facilities developed for this project enable critical currents up to 3000 A to be measured in fields up to 12 T in the presence of longitudinal or transverse stress. NIST is also involved in round robins on critical current measurements of Nb-Ti and Nb_3_Sn with both domestic and foreign participants. Calibration techniques developed for the Nb-Ti study were used in the Nb_3_Sn study. It was found that a small change in the mounting technique could result in a 40% change in the critical current density at 12 T. Mandrel material and geometry were also a source of error [154, 155].

The problem of current ripple on critical current measurement was studied [156, 157]. Ripple (the periodic departure from a dc output level) reduces the measured dc critical current, *I*_c_, and causes noise at the input to the voltmeter used for measurements. A theoretical model of rippling was developed which was in good agreement with the experimental data and can be used to estimate the effects of current ripple on the measured dc *I*_c_. It was also found that the effect of ripple should scale with its fraction of the *I*_c_, and will depend upon the shape of the V-I curve.

At present, the material of choice in the windings for magnets is Nb-Ti. The effect of stress on current degradation has been studied by Ekin et al. Current degradation as a function of strand location and field angle on cable compacted into a keystone shape was evaluated [158]. It was found that cabling can lead to localized reductions in *I*_c_ within a single strand. The widest spread in local *I*_c_ along the cable strands was with the field perpendicular to the cable edge. Unfortunately, in the dipole magnet orientation, this orientation is near the critical orientation. The relevant *I*_c_ criteria may be a spatial average (the strand *I*_c_). Therefore, both magnetic-field orientations, perpendicular and parallel to the cable width, need to be tested for *I*_c_ [159]. In addition, a large difference in current carrying capacity can exist between thick and thin cable edges, and thus, changing the direction of the test current can affect the measured *I*_c_.

**Figure 30:**
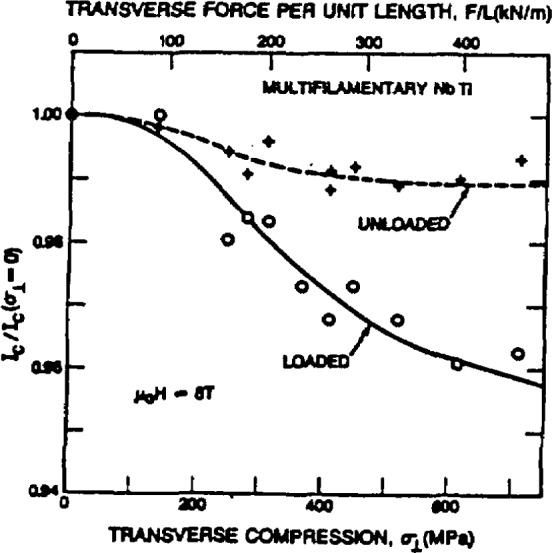
Effect of transverse compression force on the critical current of a NbTi conductor [160].

As illustrated in figure 30, the effects of various types of stress on *I*_c_ at 4 K also were studied [160]. It was found that *I*_c_ degradation from transverse compression was much less than from axial tension in terms of overall conductor stress but comparable in terms of stress on NbTi filaments. More stress can be developed in axial tension than in transverse compression because of the matrix. *I*_c_ is 95% reversible for both stresses indicating that the effects of stress will be seen only when the conductor is under stress. The primary source of degradation is a stress-induced reversible decrease in *H*_c2_. It was found [161] that the effect on the critical current is independent of the temperature at which the stress is applied. Existing data obtained at 4 K can therefore be used to determine the degradation of *T*_c_ arising from room-temperature fabrication stress, cool-down stress, and 4 K stress due to the Lorentz force when the magnet is energized. Coupling losses in multifilamentary NbTi wire were studied [156] by vibrating sample magnetometry. Losses for wires with long twist lengths were up to twice the hysteresis losses. Using short twist lengths reduced these losses.

**Figure 31:**
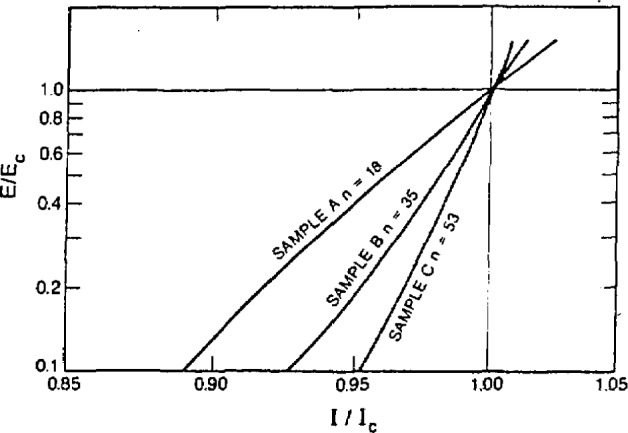
Logarithmic plot of electric field vs current for NbTi samples with different *n* [152].

Non-uniformity of sample diameter (sausaging) of the filaments also degrades performance [152]. Sausaging causes a change in E-I response resulting in a significant electric field below *I*_c_ leading to heating and decreased stability. In the relationship *E* α *I*”, the value of *n* is related to the degree of sausaging with smaller values of *n* implying more necking. Therefore, the value of n can be used to estimate filament regularity (fig. 31).

Another low-temperature superconductor which can be used for magnet applications is Nb_3_Sn. A study of the effect of transverse stress on *I*_c_ degradation showed that the intrinsic effect on the upper critical field is about 10 times that of axial stress [162, 163]. This effect scales with conductor thickness and as a result places limits on conductor dimensions and the spacing between distributed reinforcements in large magnets. This is important in applications calling for larger conductors needed to limit inductance and keep induced quench voltages low in large magnet applications. Stress concentration at strand crossover points can significantly enhance the effects. This effect is reversible, but not totally. Hysteresis losses were measured [156] on a series of fine filament Nb_3_Sn superconductors made by the internal tin process. Hysteresis was measured as a function of filament diameter and interfilament separation. Losses were greater than predicted. This was due to interfilament bridging across the wires. The critical interfilament separation, for which the critical-state model would be accurate, was determined.

The cable matrix can also play a role in improving performance. The addition of manganese to a copper matrix of fine filament Nb-Ti wire was investigated by Goldfarb et al. [164]. Manganese additions had been shown to reduce proximity-effect coupling between closely-spaced filaments [165, 166]. The investigation found that as long as the manganese content was less than 4%, there were no adverse effects.

**Figure 32:**
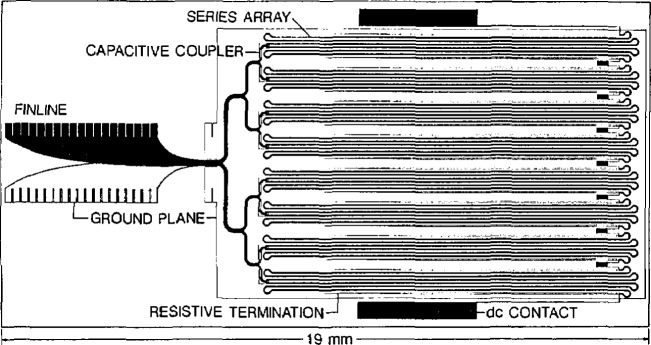
The layout for a Josephson junction array voltage standard chip fabricated using a seven-level photolithographic process [167].

NIST has developed a wide variety of applications of superconductor electronics (which will be the subject of a future review). The most successful devices that NIST researchers have produced are array voltage standards (see fig. 32) containing as many as 19,000 Josephson junctions [167]. Such integrated circuits made at NIST using VLSI techniques are already in use in most national standards laboratories around the world and in two U.S. companies. Other devices made at NIST are ultra-high-speed analog-to-digital converters, superconductor-insulator-superconductor mixers for radio astronomy at frequencies up to 300 GHz, SQUIDs with sensitivities approaching the uncertainty principle limit, samplers with response times of less than 10 ps, counters with rates above 100 GHz and sensitivity to pulses of 10^−18^ J, and an ultra-sensitive microwave and infrared detector based on the kinetic inductance of very thin superconducting films.

## 14. Conclusion

This review paper has attempted to show the breadth of NIST’s work in superconductivity. Major contributions to the materials science, standardization, and engineering applications of superconductors are evident. To maintain a reasonable length, many topics have not been covered in the depth they deserve. Some of these, e.g., superconductive electronics, will be the subjects of future review articles. With all the world-wide attention on the new high-temperature superconductors and their potential economic impact, we can anticipate that NIST personnel will continue to make new and important contributions to this exciting field.

## Figures and Tables

**Table 1:** Selected superconductor applications

Application	Advantage	Comments
Generators with superconducting wires in rotors	Lifetime cost savings up to 40%	U.S. built a 10 MW prototype; USSR is building a 300 MW prototype
Energy storage rings	Efficient, site independent, can revert from charging to discharging mode in less than 1 second. Can stabilize system	Pilot programs
Power transmission lines	Reduced resistive losses	Prototypes tested
Magnets for magnetic resonance imaging	Superconducting magnets result in shorter exposure times and sharper images compared to conventional magnets	Largest current commercial application of superconductivity; wide acceptance as medical diagnostic tool
Chip interconnects	Lack of electrical resistance; reduces heat buildup; permits dense packing…rapid transmission of signal	In research stage
SQUIDS (superconducting quantum interference devices)	Extremely sensitive to magnetic fields	Used for mineral exploration; anti-submarine warfare potential; development underway for use in medical diagnosis
Josephson Junction switches	Fast switching times, low power dissipation, dispersionless transmission	Used in fast-sampling oscilloscope; potential computer logic elements
Josephson Junction voltage standards	Reliable, stable. Absolute voltage based on fundamental constants	In use by, and available from, NIST
Magnets for fusion devices	Magnets to confine plasma	Prototype systems constructed in France and the Soviet Union
High-energy physics	High fields to guide beams, reduced energy consumption	Superconducting supercollider
Ship propulsion (motors)	Smaller, quieter motors; elimination of gearbox	Navy has protoype
Magnetohydrodynamic (MHD) power generation	High fields interact with a plasma to generate electricity	Prototypes constructed by the Soviet Union
Magnets for MHD ship propulsion	Quiet, more efficient, higher potential speeds	Demonstrated by Japan
Magnetic casting	Eliminates contamination	
Magnetic separation	Separates weakly magnetic materials	
Magnetic bearings	Eliminates friction	
IR sensors	Smaller packages	
Magnets for magnetically levitated trains (MAGLEV)	Rapid and efficient mode of transportation	Demonstrated by Japan
